# GePI: large-scale text mining, customized retrieval and flexible filtering of gene/protein interactions

**DOI:** 10.1093/nar/gkad445

**Published:** 2023-05-24

**Authors:** Erik Faessler, Udo Hahn, Sascha Schäuble

**Affiliations:** Jena University Language and Information Engineering (JULIE) Lab, Friedrich Schiller University Jena, Fürstengraben 30, 07743 Jena, Germany; Jena University Language and Information Engineering (JULIE) Lab, Friedrich Schiller University Jena, Fürstengraben 30, 07743 Jena, Germany; Jena University Language and Information Engineering (JULIE) Lab, Friedrich Schiller University Jena, Fürstengraben 30, 07743 Jena, Germany; Microbiome Dynamics, Leibniz Institute for Natural Product Research and Infection Biology (Leibniz-HKI), 07745 Jena, Germany

## Abstract

We present GePI, a novel Web server for large-scale text mining of molecular interactions from the scientific biomedical literature. GePI leverages natural language processing techniques to identify genes and related entities, interactions between those entities and biomolecular events involving them. GePI supports rapid retrieval of interactions based on powerful search options to contextualize queries targeting (lists of) genes of interest. Contextualization is enabled by full-text filters constraining the search for interactions to either sentences or paragraphs, with or without pre-defined gene lists. Our knowledge graph is updated several times a week ensuring the most recent information to be available at all times. The result page provides an overview of the outcome of a search, with accompanying interaction statistics and visualizations. A table (downloadable in Excel format) gives direct access to the retrieved interaction pairs, together with information about the molecular entities, the factual certainty of the interactions (as verbatim expressed by the authors), and a text snippet from the original document that verbalizes each interaction. In summary, our Web application offers free, easy-to-use, and up-to-date monitoring of gene and protein interaction information, in company with flexible query formulation and filtering options. GePI is available at https://gepi.coling.uni-jena.de/.

## Graphical Abstract

**Graphical Abstract:**
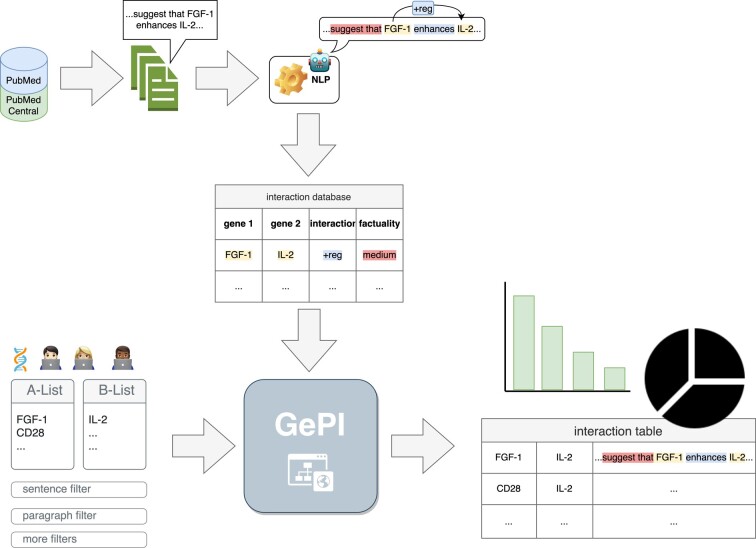

## INTRODUCTION

The molecular interactions between genes or gene products (e.g. exemplifying gene binding or positive regulatory events) are the subject of a large body of scientific research that is rapidly growing and typically communicated in scientific papers. Manually curated interaction databases such as Hprd (1), IntAct (2) and BioGrid (3) contain millions of interactions extracted from thousands of publications. Such databases offer structured, high-quality, and detailed interaction data enabling continuous scientific hypothesis testing and discovery. However, only a small fraction of the complete set of documents in PubMed (PM) (with over 35M citations) and PubMed Central (PMC) (with more than 5.1M full texts in its Open Access (OA) subset, as of March 2023) is covered by such databases through manual curation efforts. Therefore, automatic means of harvesting such information from the biomedical literature on a larger scale became a focus of research in the recent years.

String (4–6), because of its long-term development history dating back to the beginning of this millennium (7), is one of the most popular tools in the biomolecular community for gene and protein interaction retrieval and features text mining as one of its data input channels. Researchers mainly from the field of natural language processing (NLP) approached the automatic analysis of biomedical literature in a series of challenge competitions, where tools for the extraction of molecular events were rigorously evaluated against shared benchmark datasets (for a survey, see (8)). Some of the front-runner systems were subsequently integrated into Web servers, such as BioContext (9) or EvexDB (10).

We here introduce an alternative NLP-based system, Gene and Protein Interactions (GePI), for the quick and versatile retrieval of up-to-date molecular interactions from the biomedical literature contained in PM and the OA subset of PMC. GePI comes as a Web application with a graphical user interface that allows easy access also to non-programming end users. It can be queried with one or two (unlimited) *lists* of gene/protein IDs and related entity classes. Boolean full-text queries can be specified to filter for the sentence or paragraph context in which interactions occur. Additional filter options for restricting search to document titles and section headings provide further options to constrain the retrieval of gene/protein associations. These *full-text filters* can also be used without the necessity to provide input genes at all. An assessment of the degree of *factuality* (11,12) for each interaction or event is derived from verbatim signals in the documents where authors express their (un)certainty for an observed or stipulated interaction (using hedging expressions such as ‘maybe’ or ‘[our data] suggest that’). Such factuality tags may be used, e.g. to filter out negated or low-certainty interaction statements.

As a response to the often raised request for recency of information, GePI includes automated procedures for *updating* the indexed literature from PubMed and PMC multiple times a week. The obtained results are shown in a dashboard which includes base statistics and visualizations, as well as a comprehensive downloadable table summarizing all identified interactions and their associated literature passages.

## BACKGROUND

The retrieval of bio-molecular interactions from scientific documents requires gene recognition (GR), where spans of text that correspond to names or identifiers for gene entities are identified, e.g., *Arp5* as a gene in the text passage *‘[...] the protein level of Arp5 was markedly reduced [...]’*. To uniquely identify gene mentions, database IDs are assigned to them in the gene normalization (GN) step. A number of tools handle both tasks, e.g., Gnat (13), GeNo (14), GNormPlus (15), the system proposed in (16) and others. After gene and protein mentions have been recognized, semantic relations between pairs of genes/proteins must be identified—the correlate of factual assertions in documents. Again, NLP community challenges were the drivers for a number of powerful relation extractors, such as Tees (17), JReX (18), BioSem (19), Verse (20) or DeepEventMine (21).

For the choice of GePI components, we evaluated existing NLP solutions with focus on the following criteria: open source availability, performance, integratability into our NLP infrastructure, execution speed, and currency of the employed databases for GN. Considering the optimal combination of these requirements, for *gene recognition and normalization*, we equipped GePI with GNormPlus, a freely available tool that can be applied to arbitrary documents, both abstracts and full texts. It was already applied to and evaluated on the whole of PubMed and PMC in PubTator central (22), demonstrating its large-scale applicability. The tool has been continuously updated since its first release, thus ensuring its currency. Using the same assessment criteria from above, for *event extraction*, GePI runs BioSem. It showed competitive overall performance in major Shared Tasks, with very high precision values, resulting in a high fraction of relevant events. Given the requirement of correctly identifying both gene occurrences and their interactions, this ensures that GePI generates meaningful results, thus minimizing the number of false positives.

## METHODS

### Text analytics: extracting gene/protein interactions

**Figure 1.**
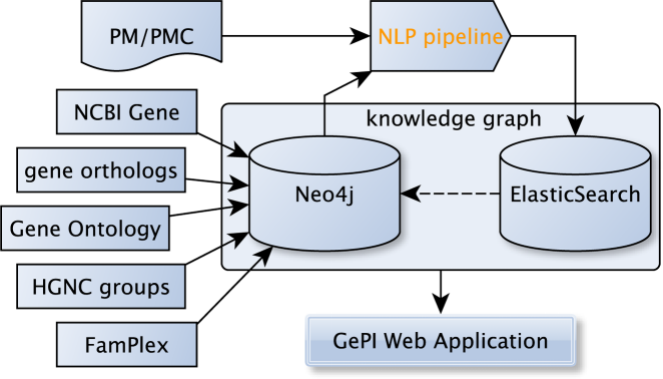
GePI components and their data flow dependencies.

Figure 1 depicts the components of the GePI application ecosystem. The input to the pipeline are XML documents from PubMed and PMC. Mentions of genes, gene products, families, protein complexes and molecular events between those entities are extracted by an NLP pipeline (depicted in Supplementary Figure S1) and indexed into an ElasticSearch (ES) cluster. A Neo4j graph database stores structured gene information that includes relationships expressing orthology, family or group membership, protein complexes and their subunits, and gene ontology annotations. This information (taken from FamPlex, GO, NCBI Gene, etc.) is incorporated in the NLP pipeline for the resolution of ambiguous gene groups, families and protein complexes. The events and interactions in ES are connected to the entities in Neo4j via unique IDs. Together they form a knowledge graph that feeds the GePI Web application. To ensure high result reliability, GePI restricts molecular interactions to occur in single sentences. Finally, the Web application leverages the gene database in Neo4j (holding stable terminological background knowledge) and the ES index (holding the continuously harvested results of relation extraction) to serve user requests. More detailed descriptions of the NLP pipeline and our data model can be found in Supplementary Material S1. An evaluation report on the gene interaction extraction components is provided in Supplementary Material S2. Evaluation results are shown in Supplementary Tables S1 and S2.

### Gene/protein search: finding molecular information

Input to the GePI interface is provided by a query form; the results of query processing are based on all interactions stored in the knowledge graph (its current size is depicted in Table 1).

**Table 1.** Current numbers of molecular interactions in GePI. This includes events with one and two arguments

	Interactions	Unique interactions
PubMed	14 441 503	411 500
PubMed Central	52 144 387	1 032 377
PM+PMC	66 585 890	1 204 986

The query form provides two input panels for lists of genes and related entities we refer to as *A*-list and *B*-list, respectively. Further input fields allow the specification of a diverse set of filters described below. Input to the *A*- and *B*-lists may consist of Ncbi Gene (23) IDs and symbols, UniProt (24) IDs, FamPlex (25) protein family and complex identifiers or names, Hgnc (26) gene group names, or Gene Ontology (GO) (27) terms. Family and group query items will include their members in the result, whereas GO term queries will include genes that have been annotated with the respective terms. Specifying only *A* items will result in an *open search*. This mode retrieves gene and protein interactions between the entries of *A* and any other interaction partners from the interaction database. If a second set of gene/protein identifiers is entered for the *B*-list, a *closed search* is performed. This mode yields only interaction items that have an element of *A* as one and an element of *B* as their other argument.

Adding genes to *A*- and *B*-lists in a search query is optional. If left out, the result of such a ‘full-text-only’ search comprises all molecular events in the interaction database that match the respective query filters. This query type can be used to identify published molecular interactions associated with specific filter terms, such as a condition (e.g., ‘elderly’) or a disease (e.g., ‘obesity’) without restrictions on the associated genes.

The query form offers two input fields to specify full-text queries either on the sentence or paragraph level. They act as context-sensitive query filters for interactions. Sentence-level context can be used to specify filter terms occurring in addition to an interaction in a sentence. The paragraph level widens the textual window around the interaction statement and allows to retrieve interactions based on relevant context keywords beyond sentence boundaries. Both filter queries can be connected by Boolean *AND* or *OR* operators. Another full-text filter is sensitive to document titles and section headings and can be applied to restrict the gene/protein interaction search to, e.g., ‘Results’ sections only, since ‘Introduction’ and ‘Background’ sections commonly refer to established (and thus less interesting) prior knowledge. Further filter options may restrict the search results to specific organisms, interaction types or factuality levels.

After the retrieval process, GePI results are presented in a dashboard. This includes aggregated overview panels and a table of complete primary data where each retrieved interaction is listed individually within its document context. The aggregation panels summarize the publication state with respect to the input and include frequency-sorted pie and bar charts of interaction partners and Sankey diagrams revealing interaction frequencies. We distinguish two types of Sankey diagrams. The first shows the most frequent interactions in the retrieval result. The second summarizes second-degree interactions, offering information on interaction partners occurring in more complex interactions than provided in the output table. Finally, the table panel provides a list of direct associations between genes or proteins associated to the current query. It discloses detailed information about the gene mentions in the text, the Ncbi Gene IDs and symbols they were mapped to, and the sentence in which interactions occurs. Where applicable, the table offers links to the source databases of the molecular entity, e.g. Ncbi Gene for genes or Hgnc for gene groups to quickly obtain full gene names, gene descriptions and further resources.

The primary data table can be downloaded as an Excel workbook, including query details and the resulting interaction data. It also lists the occurrence counts of the gene or protein symbols that take part in the interactions and how often each symbol was found in interaction with another symbol. Thus, the Excel workbook documents the query, the query result, and additional statistics enabling efficient downstream analysis. It can also be used for the manual curation of results, e.g. further filtering or the removal of erroneous result items.

## USE CASES INVOLVING GEPI

We prepared two use cases that feature GePI’s search and filtering facilities. The first one presents a search for interaction information about specific marker genes in the context of the devastating invasive pulmonary aspergillosis disease caused by the fungal opportunistic pathogen *Aspergillus fumigatus* (see Supplementary Material S3) (28). The second one describes the application of GePI to investigate the Ferritin H chain in the context of disease tolerance in sepsis (see Supplementary Material S4) (29). A detailed walk-through how to use data from these studies with GePI is provided in Supplementary Materials S5 and S6. The description of the second use case includes a verification step of GePI results leveraging String (see Supplementary Data S7 for the raw protein pair confidence values given by String). We also specify the inputs to and outputs from GePI in both use cases for reference in Supplementary Data S8.

## DISCUSSION

Automated molecular event extraction from scientific literature is an area of active research to by-pass large coverage gaps in manually curated life science databases. String, for instance, brings together a multitude of biological infrastructure resources, including curated interaction and genome sequence databases. One of String’s seven information channels is concerned with textual data accessed from PM and the PMC OA subset. String’s text mining facilities collect information for the interaction of protein pairs in two fundamental ways. Firstly, statistical methods are leveraged to find over-represented proteins in documents with respect to the user input. This is used to calculate an aggregated measure of confidence that a unique pair of proteins is associated in general, instead of identifying explicit association descriptions of a protein pair in individual documents (5). This can be witnessed in String’s text mining viewer where at least two input proteins are highlighted in each citation but are not necessarily mentioned in any interaction. Secondly, a modern NLP approach is used to find protein pairs explicitly described to physically interact, i.e., to build protein complexes (5). For such cases, the text mining viewer shows explicit verbalizations of physical interactions in PM abstracts.

Unlike String, GePI is an exclusively NLP-driven tool designed towards the identification of interacting gene/protein pairs or events involving single genes/proteins from biomedical documents. As a safe-guard mechanism, GePI supplies the text passage provided with each result item. By design, GePI cannot render interaction data potentially (only) stored in structured tables, e.g. Supplementary data, external of the publication text. GePI covers a broader range of interaction types, including binding (corresponding to String’s physical interaction), regulation and activation, thus substantially widening the scope of high-quality interaction descriptions extracted from the literature. GePI’s additional capabilities of searching for a closed pair of gene lists and filtering by document context, make GePI a complementary tool to String, which (unlike GePI) processes several types of structured resources (databases and terminologies). While String calculates confidence scores aggregated from a diverse set of resources for unique protein pairs, GePI’s focus lies on the high-quality extraction of interactions explicitly described in publications together with factuality ratings extracted from the textual context of interaction descriptions in the form of hedging expressions.

In this regard, GePI is much closer in spirit to BioContext and EvexDB. However, BioContext and EvexDB allow only single gene queries to identify their interaction partners. Even worse, the BioContext Web server became offline in the meantime. EvexDB allows for queries of single genes or a pair of genes. It provides interaction information including statistics and interaction members, as well as text snippets of the identified interactions grouped by interaction type (regulation, binding, etc.). To the best of our knowledge, EvexDB has not been updated since 2013 and thus features mostly outdated knowledge. Neither tool offers further context filtering, nor non-programmatic means to search for more than two genes at once.

GePI’s value for the scientific community is also evidenced by the usage of prior versions of its NLP pipeline for a variety of experimental studies. For these studies, we developed NLP modules (30) that allow us to extract molecular event information from the whole of PM and PMC (OA subset) at any given point of time. Previous instances of our NLP pipeline were already successfully applied to identify potential interaction partners of proteins found in phosphoproteomics experiments including the 5’ adenosine monophosphate-activated protein kinase (AMPK) complex (31). By using full-text filter capabilities, we identified interactions between members of the Akt family and pyruvate dehydrogenase kinase 2 (PDK2) in the context of cellular stress (32). We also used our NLP engine to gain literature-based insight into the current state of potential interaction partners on kinases of the PI3K/Akt signaling network based on quantitative phosphoproteomics as input for our NLP service (33). We furthermore combined results from our event extraction system with large-scale gene expression analysis and multiple validation experiments to generate a molecular signature of the activation of aryl hydrocarbon receptor (AHR) (34). Finally, our efforts supported the analysis of peripheral blood samples of children to investigate childhood asthma (35).

The above mentioned usage scenarios demonstrate the flexible applicability of our NLP engines and their potential to complement life-science data set analysis and hypothesis generation. Besides its core functionality, the recognition of gene and protein interactions in huge literature repositories, GePI excels with flexible options for expressive query formulation and a wide range of filtering functions to constrain its result sets which can then be channeled to its end users by simple-to-(re)use reporting devices (Excel sheets). Furthermore, GePI operates on up-to-date textual data (based on short-term update cycles in the range of few days from the current date) and runs efficiently (as evidenced by its timely responses).

Despite the added value offered by GePI, there is also room for improvement. On the preprocessing level, BioSem was used for its superior specificity and processing performance but has been equalized, in the meantime, by deep learning-based approaches (DL) that might identify additional interaction events, e.g., DeepEventMine. However, these DL approaches have a high demand for computational power, and frequent updates incorporating new literature can become prohibitive with updates consuming significant resources and taking prolonged periods of time. Despite the focus on high precision of GePI’s NLP components, false positive results cannot be ruled out. For further result verification we recommend to query curated databases, e.g. BioGrid, or comparison of GePI results with those of String, given the same query. Indeed, we see a clear complementary relation between GePI and String: The interactions explicitly described in the literature found by GePI can be corroborated with String’s diverse set of evidence channels to obtain a high-certainty list of interactions for each of which exists a directly linked literature support. We also recommend to investigate specific text portions provided by GePI for interactions not found by BioGrid or String. If their relevance is confirmed, these may feature interactions currently not included in the other databases.

## CONCLUSION

We introduced GePI, a Web application for the fully automated extraction of molecular events from the biomedical literature. The automatically populated and updated knowledge graph includes interactions mined from the whole of PubMed and PubMed Central OA subset with regular updates to keep up with newest developments in the literature. We presented powerful query and filtering options for contextualized retrieval of interactions between genes, proteins, families and protein complexes. The interaction results can be downloaded as an Excel workbook that contains all identified relation pairs and statistics about the frequency of the interaction partners and the interactions themselves. For each result interaction, its textual source is provided for cross-checking. The NLP pipelines we presented here and their accessibility via a Web application opens our NLP service to the broad life science community to effectively foster new scientific discoveries.

## DATA AVAILABILITY

GePI is freely available at https://gepi.coling.uni-jena.de/.

## Supplementary Material

gkad445_Supplemental_Files
