# Supplementary material for: GePI: large-scale text mining, customized retrieval and flexible filtering of gene/protein interactions
Source: Nucleic Acids Res. 2023 May 24;51(W1):W237–42. doi: 10.1093/nar/gkad445 (PMC10320155; doi:10.1093/nar/gkad445)
Supplement: gkad445_Supplemental_Files [file gkad445_supplemental_files.zip › Supplementary Material S1-S6.pdf]

S1 Supplementary Material: Technical details

S1.1 NLP Pipeline

In order to populate the ElasticSearch (ES) index for the Gene and Protein Interactions (GePI) system, PubMed (PM) and PubMed Central (PMC) Open Access (OA) documents undergo a series of processing steps. These steps include the recognition and extraction of molecular events between genes or proteins in any given sentence of an input text. We additionally assemble and assign unique database identifiers from Ncbi Gene to gene and protein mentions via gene normalization (GN). These serve to create canonical links between the entries of the Ncbi Gene database and the literature and allow for efficient resolution of lexical variance and semantic ambiguity of gene and protein names.

As depicted in Supplementary Figure S1, the natural language processing (NLP) pipeline encompasses components for basic text segmentation and syntactic analysis tasks, as well as semantic analysis for genes and gene products. Entities tagged as gene/protein families, groups or protein complexes are omitted by the original GNormPlus since they cannot be grounded in databases that only contain records for individual genes like Ncbi Gene. However, such entities may participate in the description of molecular interaction events in the literature. Hence, we keep those entity mentions for grounding in FamPlex, a resource of gene and protein families and complexes [1], and the HGNC gene group database [2], see Gene Normalization.

BioSem is then used to extract events/interactions between the previously identified entities that appear in the same sentence. In this context, an event describes a dynamic process involving one or more biological entities, like genes and proteins, entailing change to the participants of the event [3]. In their original form, the output events are represented as tree structures where molecular events can also be arguments of other events. To facilitate querying, display and statistical description of the interaction data, complex events are flattened in an additional pipeline component by collecting all genes in the tree into a list. The list is further broken down into combinations of argument pairs that consist solely of gene or protein mentions. Consequently, GePI's interaction database consists of index terms that have at most two gene or protein interaction partners. Textual molecular event descriptions may also consist of a single

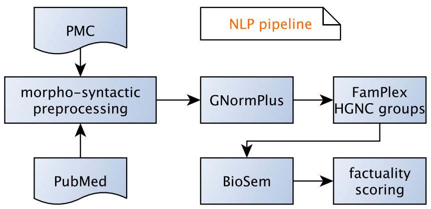

Supplementary Figure S1: Schematic design of GePI's NLP pipeline.

gene argument (e.g., *HS1 phosphorylation*). These are included in GePI by assigning a surrogate item as the second event argument. In the last step, the extracted events are sent to ES for indexing.

This pipeline is applied automatically to PM and PMC updates several times a week to offer up-to-date search of the molecular interaction literature. Its components are available in the JCoRe [4] component repository including a wrapper component for our GNormPlus adaptation that is available at `https://github.com/JULIELab/gnormplus`.

S1.2 Gene Normalization

For the unambiguous identification of genes and gene products in the processed literature we employ the gene normalization tool GNormPlus [5]. Its task is to assign a unique identifier from a gene/protein database to each gene mention in the document texts (a process termed *mapping* or *grounding*). We use the NCBI database as a canonical name space repository for GePI.

The normalization step is crucial for the core functionality of GePI—querying gene/protein interactions by lists of gene identifiers. The effects of the normalization are two-fold. On the query side, the lexical variability (ambiguity) of gene and protein names is neutralized by assigning a unique identifier. The gene ID serves as a single point of reference independent of the actual lexical form a gene or gene product was referred to in the text. It is therefore not necessary to specify synonyms as input of GePI to capture all occurrences of a gene or protein in the literature. On the document processing input side, gene normalization resolves ambiguity that arises from homonymy. Some gene or protein names denote multiple, separate items in gene and protein databases. For example, the name *ARP5* is a synonym for the Ncbi Gene symbols *ACTR3*, *ANGPTL6* and *APOBEC3C*. Dependent on the document context, the ID of the gene that was most probably referred to by the authors must be assigned. To this end, GNormPlus takes into account long forms of abbreviated gene names as introduced in the text, collects all names of genes in the document and ultimately employs an inference network to score the eligible gene candidates for each gene name in the document and picks the top-scoring one [6].

In its original form, GNormPlus recognizes mentions of individual genes and their products, as well as groups or families of them. However, due to its adaptation to GN tasks, family names are discarded. Regarding molecular interaction extraction, however, families and protein complexes might be reasonable arguments of textual statements in the literature. Because of their importance for the description of biomolecular interactions, we decided to keep otherwise discarded gene and protein group, family and complex mentions. This required a few alterations in the source code that are integrated in our version of GNormPlus.

The incorporation of new biomolecular objects leads to new challenges regarding entity grounding. Common gene and protein databases like NCBI GENE or UNIPROT list the individual building blocks of protein complexes, but do not contain entries for the resulting objects. This may lead to a new source of normalization errors. For example, the 5' adenosine monophosphate-activated protein kinase (AMPK) enzyme consists of three subunits, α, β and γ. Each of those subunits is part of a family of two to three isoforms. For each of these isoforms, entries in NCBI GENE and UNIPROT exist. A common mapping mistake is to ground mentions of the AMPK complex to one of its isoforms. While this might be a partially adequate solution in some cases – the subunits are implicitly present when their complex is mentioned – a structurally more comprehensive approach would be to include a) a normalized denotation of the complex itself and b) the additional tagging of all three subunits on the same lexical unit.

These requirements have been formerly identified and led to the development of the FAMPLEX resource described in [1]. It lists gene and protein families and complexes with diverse lexical synonyms for the textual grounding of the resource entries into the literature. Additionally, isoforms, families and complexes are connected via *IS-A* and *PART-OF* relations that represent the tree structure induced by the different description levels of protein complexes. We incorporated this resource by matching the entity strings specified in FAMPLEX to entities tagged as families or protein complexes by GNORMPLUS via string matching. We mapped the leaves of the tree structures – the individual genes or proteins – to NCBI GENE and, in this way, anchored them in the text documents via the employed GN algorithm. This enables users to issue GEPI queries for protein complexes and families that otherwise would yield empty or incomplete results.

S1.3 Factuality Status of Interactions

Scientific papers on biomolecular interactions often include negation or hedging expressions that express a restriction in the likelihood that an interaction *really* took place. Words or phrases, such as *"might", "suggest", "have evidence"*, indicate a varying degree of factuality [7] regarding the statement that is modified by these expressions. The GEPI NLP pipeline integrates the hedging detection approach described in [8]. It consists of six levels of varying likelihood a molecular event described in the literature can be assigned to, ranging from explicit negation to fully trustable assertive factual statements.

S1.4 Gene Data Model

The gene data basis for GEPI is an integration of NCBI GENE, the NCBI gene ortholog file [9], FAMPLEX, gene groups from HGNC and the Gene Ontology. We use NCBI GENE as a flat list of data records, i.e., a source of IDs and symbols for each entry. The gene ortholog file assembles individual NCBI GENE records to an abstract concept of functionally similar genes across different species. Each ortholog group is materialized in our data model with relationships to

its NCBI GENE elements to enable species-independent queries. In a similar fashion, FAMPLEX organizes sets of gene records to families and complexes, in some cases across multiple levels of *IS-A* and *PART-OF* relationships. Where applicable, FAMPLEX specifies links to external resources that we used to foster an attachment to NCBI GENE. HGNC organizes genes in hierarchical groups. Each HGNC group ID, name and synonyms constitutes a single database entry with relationships to their members. Finally, we use the functional annotations from NCBI GENE to associate genes with Gene Ontology (GO) terms to obtain a fully integrated gene data model, including orthologs, families, complexes, and GO annotations.

S1.5 Interaction Database

The GEPI interaction database is realized using ElasticSearch and contains one record for each gene/protein interaction that was extracted from the literature. The records contain one or two gene/protein arguments which is the central interaction information. We stored their data model ID and the data model IDs of all directly or indirectly governing data model concepts such as orthologs, families, protein complexes, HGNC groups and GO terms. In this way, each interaction record can be retrieved by queries aiming at different data abstraction levels.

The records include the sentence and paragraph texts in which the interaction was found, as well as the headings of all sections and subsections the interaction is directly or indirectly located in. These information pieces are the basic building blocks of the full-text search and filter functionality of GEPI. Since BIOSEM works on the sentence level, there is exactly one sentence per interaction record. The paragraph text of each interaction record reflects three levels of formal grouping in texts. On the abstract level, it is either the whole abstract or, when the abstract is structured, the respective section of the abstract like *Background*, *Methods*, *Results* etc. In PMC full texts, the paragraphs are encoded explicitly in the document source format, as well as the full text sections and their headings.

Factuality information is also stored for each interaction database item. The six different likelihood tiers are encoded as integers to enable queries with "greater than or equal" semantics, returning all interaction items above or equal to a given threshold.

S2 Supplementary Material: Evaluation Report

The quality of GePI's results is largely determined by the performance of its text analytics. Most importantly, the gene recognition, gene normalization and event extraction algorithms have to produce high-quality output to maximize the number of correct interaction results and minimize false positives. Supplementary Table S1 shows the scores reached by GNormPlus on the BioCreative II (BC2) [10] and BioCreative III (BC3) [11] GN challenge test data sets and the more recent NLM Gene dataset [12].

Supplementary Table S1: Evaluation scores of GNormPlus for the GN task. Results for BioCreative II (BC2) are taken from the GNormPlus publication, the numbers for BioCreative III (BC3) and NLM Gene have been created with our own evaluation.

	R	P	F
BC2 Test	86.4%	87.1%	86.7%
BC3	45.1%	52.3%	48.5%
NLM Gene	69.9%	73.3%	71.5%

All scores have been determined by evaluations carried out for this study with the exception of the BC2 scores because the available GNormPlus gene recognition (GR) model was trained on the whole of the BC2 GN dataset including the test portion. Furthermore, the download version of the software prohibits re-training the GR model, so we could not create a train-only model. Moreover, since gene mentions from the test set were added to the GNormPlus dictionaries, a comparable evaluation of the BC2 test data is simply precluded. Hence, we report here the official evaluation performance from [5].

The performance values for BC3 in Supplementary Table S1 were obtained by first converting the original PMC XML documents of the BC3 gold test set into the BioC [13] format used by GNormPlus with our own conversion algorithm. Next, we processed the documents in BioC format, which is used to incorporate GNormPlus into the GePI production system. Thus, the displayed numbers reflect the output quality to be expected for the final system. This may also explain minor differences to the previously published evaluation numbers. There might be small deviations between our BioC conversion and the document format used for the official GNormPlus evaluation.

The second essential step is the extraction of molecular events from the literature. The BioSem software we integrated into GePI has been evaluated by the authors for the BioNLP Shared Task 2011 and 2013 challenges on event extraction [14, 15]. We could confirm the originally reported performance scores reported in [16] with our own experiments (Supplementary Table S2). We opted for using BioSem to exploit its exceptional precision scores and computational efficiency.

Supplementary Table S2: Scores reached by BioSem in the BioNLP Shared Tasks 2011 and 2013 on the respective test data.

	R	P	F
BioNLP ST11 abstracts	41.89%	69.72%	52.34%
BioNLP ST11 full texts	44.47%	66.63%	53.34%
BioNLP ST13	42.47%	62.83%	50.68%

S3 Supplementary Material: Use Case 1

Zoran et al. (2022) [17] investigated patients who suffered from invasive pulmonary aspergillosis caused by the fungal pathogen *Aspergillus fumigatus* after receiving an allogeneic stem cell transplantation. The authors investigated multiple gene expression data sets as well as the existing literature for promising targets to follow up. They identified 9 genes and their protein products that were investigated further: *CASP3*, *CD40*, *CXCL8*, *ITGB3*, *LGALS2*, *MMP1*, *MMP9*, *SERPINE1* and *VEGFA*. Using these genes as GePI *A*-list input query generates 89,786 resulting interaction events (as of March 16, 2023) with *CXCL8*, *MMP9* and *CASP3* being the genes with the most frequent interactions reported. Although this reveals huge literature support for interaction events involving these genes, downstream analysis may be hampered by the vast amount of results. In order to focus and contextualize the result, adding "stem cell transplantation" — "alloSCT" — "HSCT" — "fumigatus" as paragraph filters helped reduce this number to 65 reported events. 11 of these were associated to positive regulation, while 10 were associated to a binding event hinting at the active role of the majority of the queried genes in regulating their event partners. While *CXCL8* and *CASP3* were among the three most frequent event partners of the input gene list, *MMP9* was found only 4 times in the context of the paragraph filter, while *CD40* was reported 12 times in the required context. This highlights the need for additional context, if the aim of a search is to identify the most frequent event members. A possible follow-up query would be to check also for inflammatory relevance. Adding the strict additional requirement of "inflammation" at the sentence level and restricting the factuality level to "assertion" to the last query, resulted in 5 remaining events. Only *CXCL8* and *MMP9* were identified as event partners, while positive regulation of *MMP9* on *TGF-β1* was identified two times in the full-text of Hong et al. 2022 [18]. This exemplifies that already small input lists can generate a high number of molecular events, while further contextualization can reduce the number of identified interactions significantly. It should be noted that trying to identify the same list of molecular events and the supporting literature with a regular PM search (https://pubmed.ncbi.nlm.nih.gov/) will turn out to be much more cumbersome, since PM is not designed to search for indexed molecular events and contextualize them on either the sentence level, the paragraph level, or both.

S4 Supplementary Material: Use Case 2

In a study by Weis et al. (2017) [19], metabolic adaptation was found to establish disease tolerance in sepsis. Ferritin H chain (FTH) was required for this observation and had effects on glucose-6-phosphatase and liver gluconeogenesis, both metabolic components to transform glucose into cellular needs. Running an open query with ("ferretoxin" — "FTH") + ("sepsis" — "glucose" — "gluconeogenesis") as paragraph filter returned a list of 39 molecular events contextualized with the occurrence of ferretoxin and either sepsis, glucose or gluconeogenesis in the same paragraph. After erasing false positives, this list comprised 34 genes, including *FTH1* and also its light chain component ferritin light chain (*FTL*) among others. This gene list could, in turn, be used to run an *A*-*B* (closed) search with "sepsis" as additional paragraph filter. If using identical entries for *A* and *B* lists as in this case, a user can scan the complete literature for the most frequent, and thus, e.g., most likely, gene-gene connections and molecular events these genes give rise to in a given context (here, sepsis). This resulted in a list of 177 molecular events, where the most frequently occurring events are *HMOX1-NFE2L2*, *KEAP1-NFE2L2* and *HMOX1-TNF* that either can be analyzed as proof-of-principle in a new study, or ruled-out as a sufficient number of reports highlighted these interactions already. *TNF*, *NFE2L2* and *HMOX1* were among the genes with the most diversified event partners highlighting their potential central role in orchestrating immune reactions in the context of sepsis. These genes may thus be potential candidates to be included in a new study on sepsis.

In summary, if a process and its context is known, GePI can be utilized to search for interaction events occurring in the context of the given search filters. Subsequently, the resulting gene list can be used to look for frequently occurring molecular events between members of the identified genes. In contrast, gene-gene interactions that are not found in the given context or only with very low frequency, might pinpoint to new or underestimated gene-gene regulatory events that may also be important to include in follow-up studies in a given or novel context (e.g., sepsis).

References

[1] Bachman, J. A., Gyori, B. M., and Sorger, P. K. (2018) FamPlex: a resource for entity recognition and relationship resolution of human protein families and complexes in biomedical text mining. *BMC Bioinformatics,* **19**, #248 doi:10.1186/s12859-018-2211-5 PMCID:PMC6022344.

[2] Seal, R. L., Braschi, B., Gray, K., Jones, T. E. M., Tweedie, S., Haim-Vilmovsky, L., and Bruford, E. A. (2023) Genenames.org: the HGNC resources in 2023. *Nucleic Acids Research,* **51**(D1), D1003–D1009 gkac888 doi:10.1093/nar/gkac888 PMCID:PMC9825485.

[3] Kim, J. D., Ohta, T., and Tsujii, J. (2008) Corpus annotation for mining biomedical events from literature. *BMC Bioinformatics,* **9**, 1–25 doi:10.1186/1471-2105-9-10.

[4] Hahn, U., Matthies, F., Faessler, E., and Hellrich, J. (2016) UIMA-based JCoRe 2.0 goes GitHub and Maven Central: State-of-the-art software resource engineering and distribution of NLP pipelines. In *LREC 2016 — Proceedings of the 10th International Conference on Language Resources and Evaluation. Portorož, Slovenia, 23-28 May 2016* pp. 2502–2509.

[5] Wei, C.-H., Kao, H.-Y., and Lu, Z. (2015) GNormPlus: an integrative approach for tagging genes, gene families, and protein domains. *BioMed Research International,* **2015**, #918710 doi:10.1155/2015/918710 PMCID:PMC4561873.

[6] Wei, C.-H. and Kao, H.-Y. (2011) Cross-species gene normalization by species inference. *BMC Bioinformatics,* **12**(Suppl 8), S5 http://www.biomedcentral.com/1471-2105/12/S8/S5 doi:10.1186/1471-2105-12-S8-S5 PMID:22151999 PMCID:PMC3269940.

[7] Saurí R. and Pustejovsky, J. D. (2012) Are you sure that this happened? Assessing the factuality degree of events in text. *Computational Linguistics,* **38**(2), 261–299 doi:10.1162/COLI_a_00096.

[8] Hahn, U. and Engelmann, C. (2014) Grounding epistemic modality in speakers' judgments. In Pham, D. and Park, S., (eds.), *PRICAI 2014 — Proceedings of the 13th Pacific Rim International Conference on Artificial Intelligence. Gold Coast, Queensland, Australia, 1-5 December, 2014,* Cham: Springer pp. 654–667 doi:10.1007/978-3-319-13560-1_52.

[9] Sayers, E. W., Bolton, E. E., Brister, J. R., Canese, K., Chan, J., Comeau, D. C., Connor, R., Funk, K., Kelly, C., Kim, S., et al. (2022) Database resources of the National Center for Biotechnology Information. *Nucleic Acids Research,* **50**(D1), D20–D26 doi:10.1093/nar/gkab1112 PMCID:PMC8728269.

[10] Morgan, A. A., Lu, Z., Wang, X., Cohen, A. M., Fluck, J., Ruch, P., Divoli, A., Fundel, K., Leaman, R., Hakenberg, J., Sun, C., Liu, H. H., Torres, R., Krauthammer, M., Lau, W. W., Liu, H., Hsu, C. N., Schuemie, M., Cohen, K. B., and Hirschman, L. (2008) Overview of BioCreative II gene normalization. *Genome Biology,* **9**(SUPPL. 2) `doi:10.1186/gb-2008-9-s2-s3` `PMID:18834494`.

[11] Lu, Z., Kao, H. Y., Wei, C. H., Huang, M., Liu, J., Kuo, C. J., Hsu, C. N., Tsai, R. T., Dai, H. J., Okazaki, N., Cho, H. C., Gerner, M., Solt, I., Agarwal, S., Liu, F., Vishnyakova, D., Ruch, P., Romacker, M., Rinaldi, F., Bhattacharya, S., Srinivasan, P., Liu, H., Torii, M., Matos, S., Campos, D., Verspoor, K., Livingston, K. M., and Wilbur, W. J. (2011) The gene normalization task in BioCreative III. *BMC Bioinformatics,* **12**(SUPPL. 8) `doi:10.1186/1471-2105-12-S8-S2` `PMID:22151901`.

[12] Islamaj, R., Wei, C. H., Cissel, D., Miliaras, N., Printseva, O., Rodionov, O., Sekiya, K., Ward, J., and Lu, Z. (2021) NLM-Gene, a richly annotated gold standard dataset for gene entities that addresses ambiguity and multi-species gene recognition. *Journal of Biomedical Informatics,* **118**(March), 103779 `doi:10.1016/j.jbi.2021.103779` `PMID:33839304`.

[13] Comeau, D. C., Doğan, R. I., Ciccarese, P., Cohen, K. B., Krallinger, M., Leitner, F., Lu, Z., Peng, Y., Rinaldi, F., Torii, M., Valencia, A., Verspoor, K. M., Wiegers, T. C., Wu, C. H., and Wilbur, W. J. (2013) BioC: a minimalist approach to interoperability for biomedical text processing. *Database –The Journal of Biological Databases and Curation,* **2013**, bat064.

[14] Kim, J.-D., Pyysalo, S., Ohta, T., Bossy, R., Nguyen, N. T. H., and Tsujii, J. (2011) Overview of BioNLP Shared Task 2011. In *BioNLP 2011 — Proceedings of the BioNLP Shared Task 2011 Workshop on Biomedical Natural Language Processing @ ACL-HLT 2011. Portland, Oregon, USA, 24 June 2011* pp. 1–6.

[15] Nédellec, C., Bossy, R., Kim, J.-D., Kim, J.-j., Ohta, T., Pyysalo, S., and Zweigenbaum, P. (2013) Overview of BioNLP Shared Task 2013. In *BioNLP 2013 — Proceedings of the BioNLP Shared Task 2013 Workshop @ ACL 2013. Sofia, Bulgaria, August 9, 2013* pp. 1–7.

[16] Bui, Q. C. and Sloot, P. M. a. (2012) A robust approach to extract biomedical events from literature. *Bioinformatics,* **28**(20), 2654–2661 `doi:10.1093/bioinformatics/bts487` `PMID:22859502`.

[17] Zoran, T., Seelbinder, B., White, P., Price, J., Kraus, S., Kurzai, O., Linde, J., Häder, A., Loeffler, C., et al. (2022) Molecular profiling reveals characteristic and decisive signatures in patients after allogeneic stem cell transplantation suffering from invasive pulmonary aspergillosis. *Journal of Fungi,* **8**(2) `doi:10.3390/jof8020171` `PMCID:PMC8880021`.

[18] Hong, C., Lu, H., Huang, X., Chen, M., Jin, R., Dai, X., Gong, F., Dong, H., Wang, H., and Gao, X.-M. (2022) Neutrophils as regulators of macrophage-induced inflammation in a setting of allogeneic bone marrow transplantation. *Stem Cell Reports,* **17**(7), 1561–1575 doi:10.1016/j.stemcr.2022.05.021 PMCID:PMC9287675.

[19] Weis, S., Carlos, A. R., Moita, M. R., Singh, S., Blankenhaus, B., Cardoso, S., Larsen, R., Rebelo, S., Schäuble, S., Del Barrio, L., et al. (2017) Metabolic adaptation establishes disease tolerance to sepsis. *Cell,* **169**(7), 1263–1275.e14 doi:10.1016/j.cell.2017.05.031 PMCID:PMC5480394.

S5 Supplementary Material: Case Study 1 Demonstration

This document shows screenshots that complement the description of the case study on data from Zoran et *al*. (2022) in Supplementary Section S3.

The input list for the initial GePI query was:

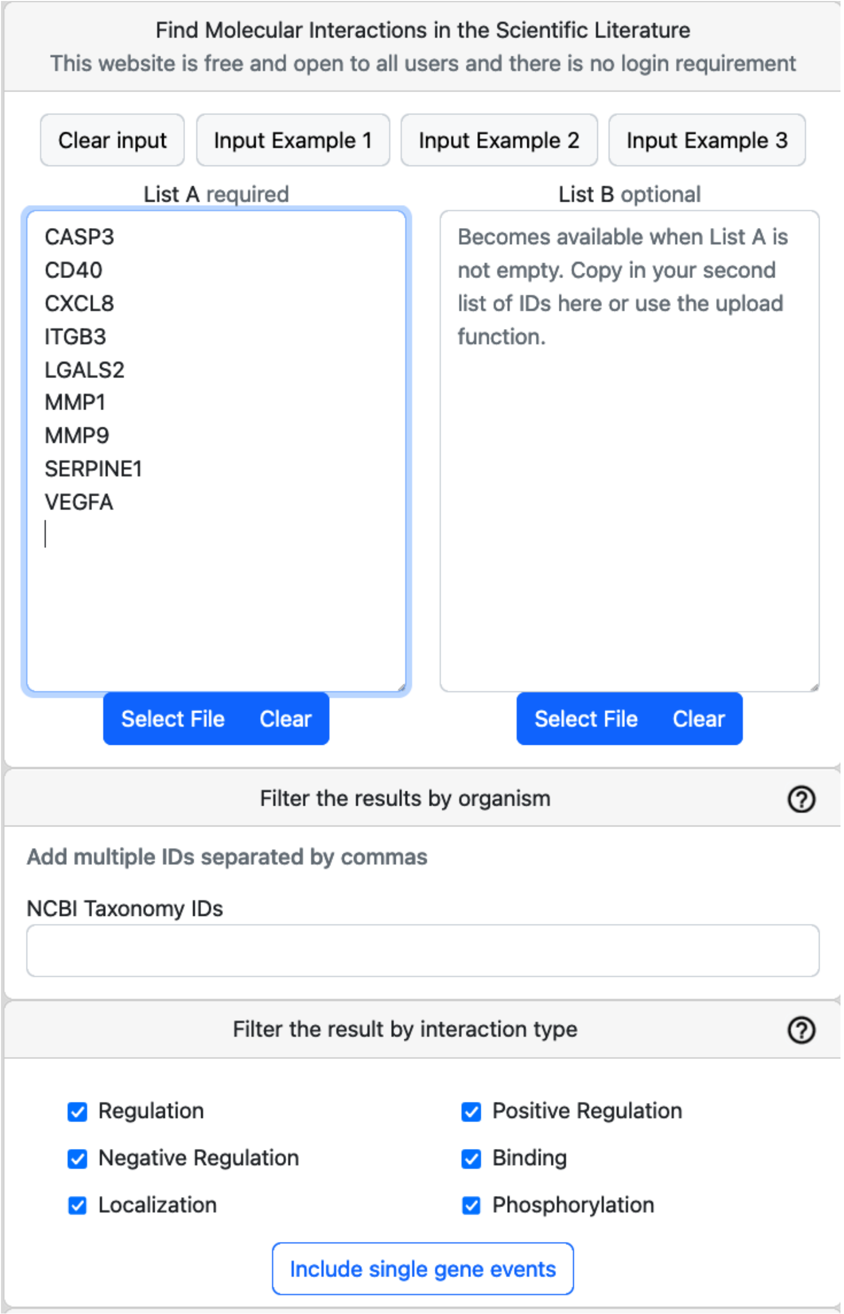

The result to this query is displayed in the following screenshots. Statistics and visualizations give a summary about the distribution of interactions and their involved genes:

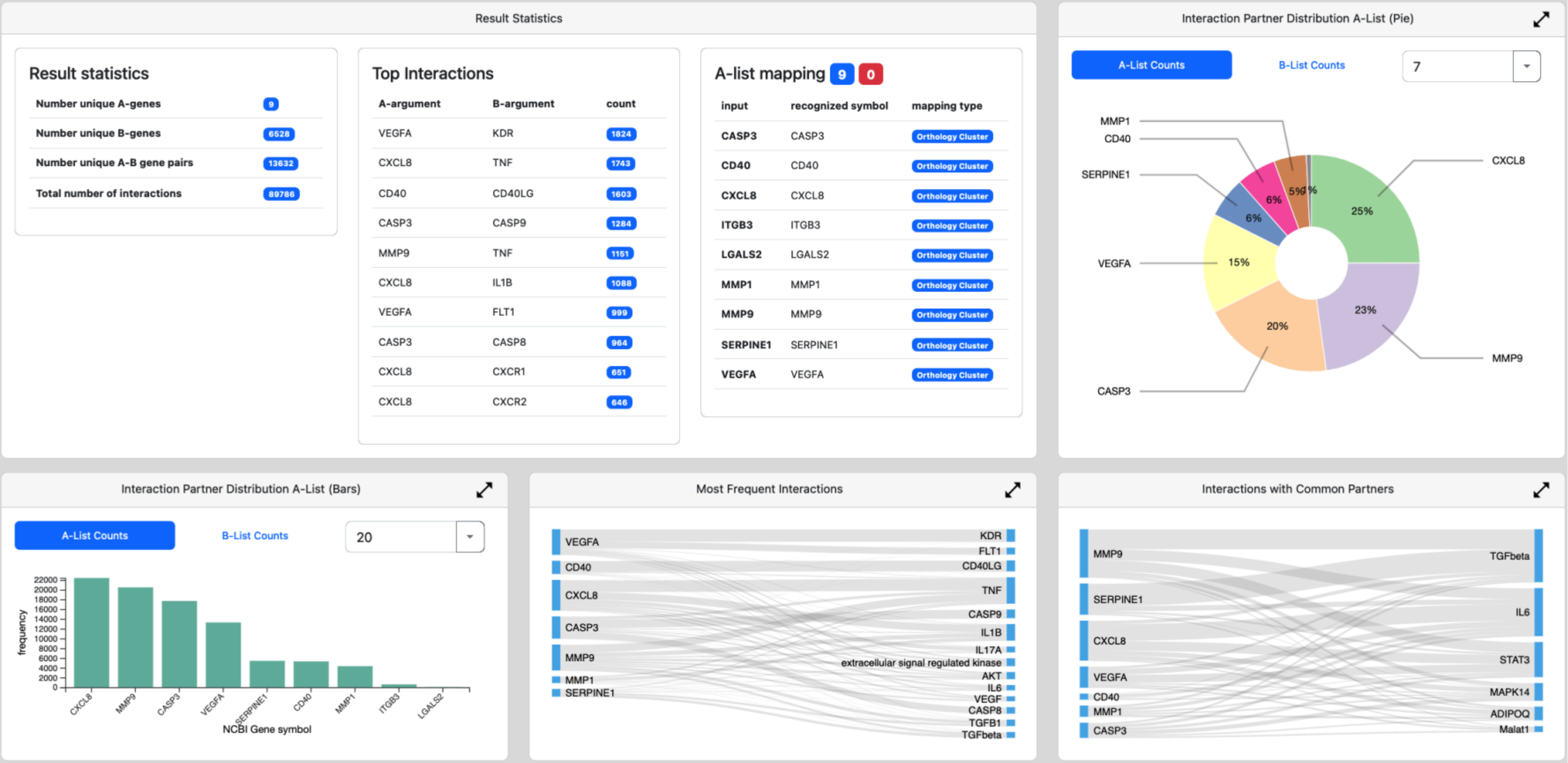

The result table lists the interactions individually with details about the recognized genes, the interaction types, the factuality assessment, the literature source and the document context. The symbols are linked to the source database of the gene (e.g. NCBI Gene, HGNC groups or FamPlex) to provide its full name and further resources:

Table View

1 2 3 4 5 6 7 8 9 10 11 ... 999 1000

Gene A Symbol	Gene B Symbol	Gene A Gene ID	Gene B Gene ID	Relation Types	Factuality	Fulltext Match Source	Document ID	Context
Casp3	Ccl4	12367	20303	Positive_regulation	★★★	none	PMC9855146	In the present study, significant upregulation of caspases-9 and -3 activities in CCl4 -treated liver tissues of mice were detected, which were partly revised by NOOT supplementation in a dose-dependent manner (Figure 6).
CXCL8	SLPI	3576	6590	Gene_expression, Regulation, Positive_regulation	★★★	none	PMC2212970	Effect of SLPI on LPS-induced TNF-α (A), IL-8 (B), and IL-10 (C) protein production by U937 macrophages.
CXCL8	PTP4A3	3576	11156	Positive_regulation	★★★	none	PMC8750532	IL-8 secreted by TAMs demonstrated their involvement in macrophage-derived angiogenesis [] whereas TAMs secreting IL-6 and IL-8 facilitated the metastasis of colorectal cancer induced by PRL-3 (Phosphatase of regenerating liver) marker [].IL-8 and MDSCs—DSCs represent an extremely heterogeneous cell population of immature myeloid cells which derive from the bone marrow hematopoietic precursors due to the alteration of myelopoiesis in pathological states as cancer or inflammation.
MMP9	TGFB1	4318	7040	Regulation	★★★	none	PMC9855939	The transcriptional level of MMP-9 is regulated by cytokines and growth factors (IL-13, TNF, transforming growth factor beta (TGF), and vascular endothelial growth factor (VEGF)), as well as epigenetic mechanisms (histone modification, DNA methylation, and non-coding RNA).
CD40	RELA	958	5970	Binding, Phosphorylation, Positive_regulation	★★★	none	PMC3656093	Engagement of CD40 by its trimerized ligand (tCD40L) leads to phosphorylation of p65 , a subunit of NF-κB (Figure 2A).
Vegfa	PI3 kinase	22339	FPLX:PI3K	Phosphorylation, Positive_regulation	★★★	none	PMC8855503	VEGF treatment enhanced the phosphorylation of PI3K , Akt and AMPK (assayed by western blotting), as well as glucose consumption and metabolism (assayed by western blotting and glucose uptake assay), in the C2C12 myotubes.
CASP3	DEFB4A	836	1673	Negative_regulation, Positive_regulation	★★★	none	PMC3658579	Similarly, hBD-3 but not hBD-1, hBD-2 and hBD-4 significantly suppressed the activation of caspase 3 (data not shown).
CXCL8	PLA2G10	3576	8399	Localization, Positive_regulation	★★★	none	PMC8962440	We also found that several human recombinant phospholipases A2 (sPLA2) (group V and group X) induced the release of ANGPT1 > VEGF-A > CXCL8 from neutrophils [].
CXCL8	ANGPT1	3576	284	Positive_regulation	★★★	none	PMC8962440	However, only ANGPT1 , but not ANGPT2, induces cytokine release (e.g., IL-1β, IL-8 /CXCL8) from human neutrophils [,].
CXCL8	IL1B	3576	3553	Binding	★★★	none	PMC3798602	The analysis between pro-inflammatory cytokines, IL-8, and CRP in IC/BPS serum samples, showed significant association between IL-1β and IL-8 (Fig. 2A , r2 = 0.54,p<0.001), IL-6 and CRP (Fig. 2B , r2 = 0.17,p =0.01), IL-6 and IL-8 (r2 = 0.17,p = 0.02), and IL-6 and TNF-α (r2 = 0.16,p =0.03).

The follow up search contained the paragraph-level filter "stem cell transplantation" | "alloSCT" | "HSCT" | "fumigatus":

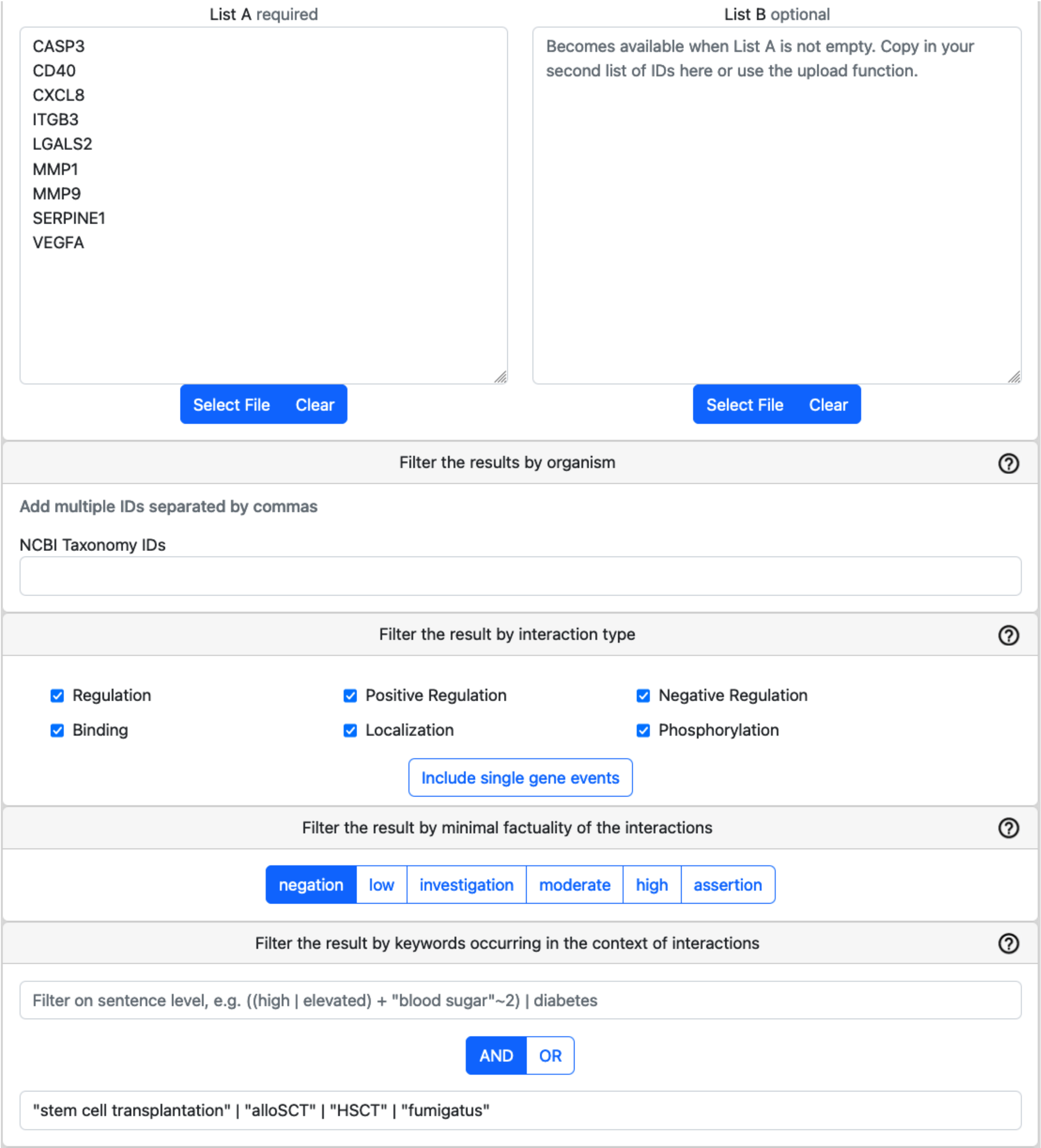

The result to the filtered query is shown blow:

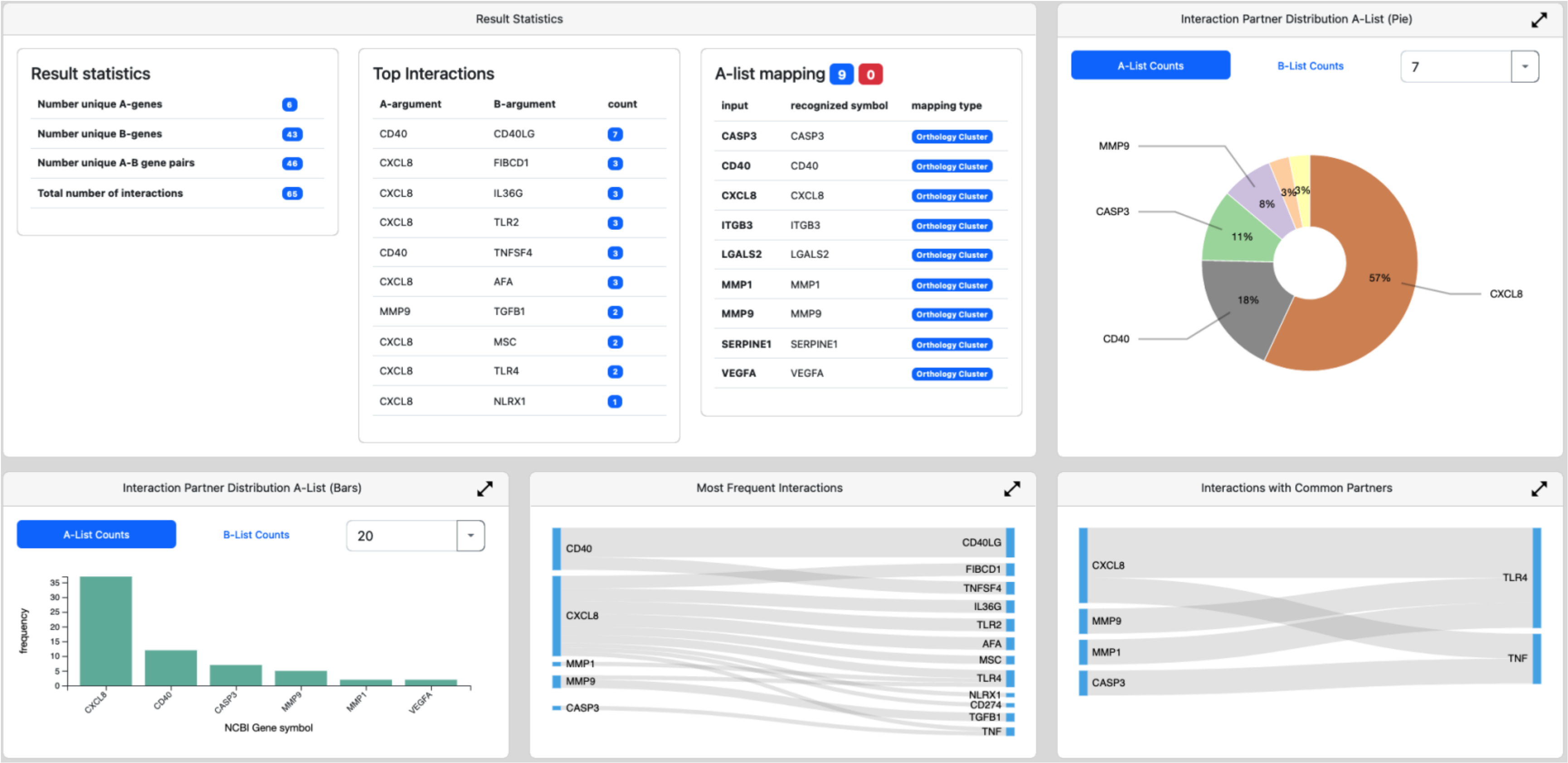
Result Statistics
Result statistics
Number unique A-genes 6
Number unique B-genes 43
Number unique A-B gene pairs 46
Total number of interactions 65
Top Interactions
A-argument B-argument count
CD40 CD40LG 7
CXCL8 FIBCD1 3
CXCL8 IL36G 3
CXCL8 TLR2 3
CD40 TNFSF4 3
CXCL8 AFA 3
MMP9 TGFB1 2
CXCL8 MSC 2
CXCL8 TLR4 2
CXCL8 NLRX1 1
A-list mapping 9 0
input recognized symbol mapping type
CASP3 CASP3 Orthology Cluster
CD40 CD40 Orthology Cluster
CXCL8 CXCL8 Orthology Cluster
ITGB3 ITGB3 Orthology Cluster
LGALS2 LGALS2 Orthology Cluster
MMP1 MMP1 Orthology Cluster
MMP9 MMP9 Orthology Cluster
SERPINE1 SERPINE1 Orthology Cluster
VEGFA VEGFA Orthology Cluster
Interaction Partner Distribution A-List (Pie)
A-List Counts
B-List Counts
7
MMP9
CASP3
CD40
CXCL8
57%
18%
11%
8%
3%
3%
Interaction Partner Distribution A-List (Bars)
A-List Counts
B-List Counts
20
frequency
35
30
25
20
15
10
5
0
CXCL8
CD40
CASP3
MMP9
MMP1
VEGFA
NCBI Gene symbol
Most Frequent Interactions
CD40
CXCL8
MMP1
MMP9
CASP3
CD40LG
FIBCD1
TNFSF4
IL36G
TLR2
AFA
MSC
TLR4
NLRX1
CD274
TGFB1
TNF
Interactions with Common Partners
CXCL8
MMP9
MMP1
CASP3
TLR4
TNF

Table View

1 2 3 4 5 6 7

Gene A Symbol	Gene B Symbol	Gene A Gene ID	Gene B Gene ID	Relation Types	Factuality	Fulltext Match Source	Document ID	Context
CD40	CD40LG	958	959	Binding	★★★	paragraph	PMC3447666	CD40 - CD40L interaction regulates several aspects of cell-mediated immunity, including activation of antigen-presenting cells, CD4+ and CD8+ T cells, and stimulation of IL-12/IFN-γ production [,].<br>SinceA. fumigatus has been shown to induce platelet activation [,], low serum sCD40L...
MMP9	CSF3	4318	1440	Localization, Positive_regulation	★★★	paragraph	PMC5380331	It was shown that G-CSF induces the release of neutrophil elastase (NE) and matrix metalloproteinase-9 (MMP-9).<br>In the case of allogenic stem cell transplantations, HSC from a healthy donor are...
CD40	CD40LG	958	959	Binding, Negative_regulation	★★★	paragraph	PMC3308063	Immunotherapies using the chimeric anti-CD20 antibody rituximab [], tocilizumab blocking IL-6 effects [], blocking CD154 - CD40 interactions [], and autologous stem cell transplantation [] in severe SLE demonstrated that the expanded memory B-cell and plasma cell populations could be normalized and resulted in predominantly naïve B cells after successful interventions.<br>...cell transplantation [] in severe SLE demonstrated that the expanded memory B-cell...
CD40	IFNA	958	FPLX:IFNA	Positive_regulation	★⯪☆	paragraph	PMC4714171	It has been suggested that IFNα may activate CD40 -ligand, a member of the tumor necrosis factor (TNF) superfamily which binds to CD40 on dendritic cells.<br>...cell transplantation.
CXCL8	TLR4	3576	7099	Regulation, Positive_regulation	★⯪☆	paragraph	PMC9151998	fumigatus, SP-D was proven to connect LPS recognition and IL-8 response through TLR4-MyD88/NF-kB pathway, the expression of which might conversely be regulated by TLR4 -Jnk activation.<br>As TLR4 inhibitors reduced the IL-1β and IL-8 production activated byA. fumigatus...
CD40	CD40LG	958	959	Binding	☆☆☆	paragraph	PMC7063425	The mutated CD40L fail to bind CD40 on B cells thus affecting immunoglobulin class switch recombination resulting in the absence of IgG, IgA, IgE with a normal or elevated IgM.<br>...autoimmunity and malignancies in some individuals., XHIM can be treated by allogenic HSCT...
CXCL8	MSC	3576	9242	Positive_regulation	★★★	paragraph	PMC6744626	The mechanistic link between inflammation and upregulation of SASP components was confirmed by experiments showing the upregulation of IL-6, IL-8, and MCP-1 in control MSC after exposure to TNF-alpha.<br>...cell transplantation.
CXCL8	NADPH oxidase	3576	FPLX:NADPH_oxidase	Gene_expression, Positive_regulation	★★★	paragraph	PMC4598756	This may be related to NADPH oxidase which is necessary for IL-8 synthesis in response to TLR activation byP.<br>...includingStaphylococcus aureus,Haemophilus influenzae,Burkholderia cepacia,Aspergillus fumigatus...
CXCL8	AFA	3576	170	Localization, Positive_regulation	★★★	paragraph	PMC3149602	In whole blood from healthy subjects and CF patients, AFA induced the release of IL-8 to 113.6±27.2 ng/mL (n = 4) and 227.1±39.7 ng/mL (n = 23), respectively.<br>...(AFA) (not shown).
CXCL8	CFTR	3576	HGNCG:309	Regulation, Localization	★⯪☆	paragraph	PMC3149602	Our results, which should be confirmed on a larger patient cohort, suggest that the CFTR genotype may be involved in whole blood IL-8 release related to systemic inflammation in CF patients.<br>...find a significant difference in IL-8 production after stimulation with LPS or AFA...

The last query in this use case added the "inflammation" sentence filter and set the event factuality to "assertion":

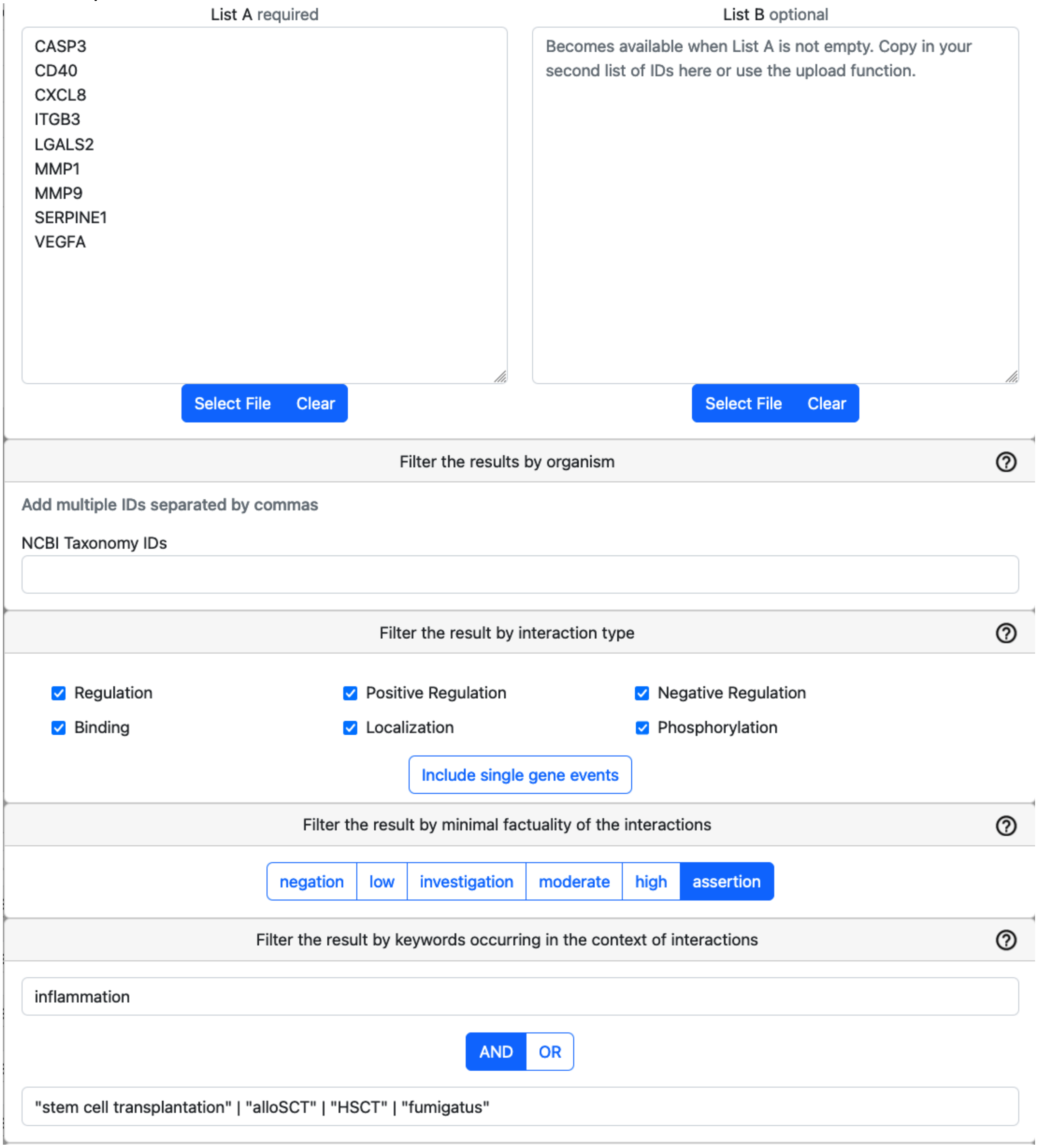

Which produced this result shown below:

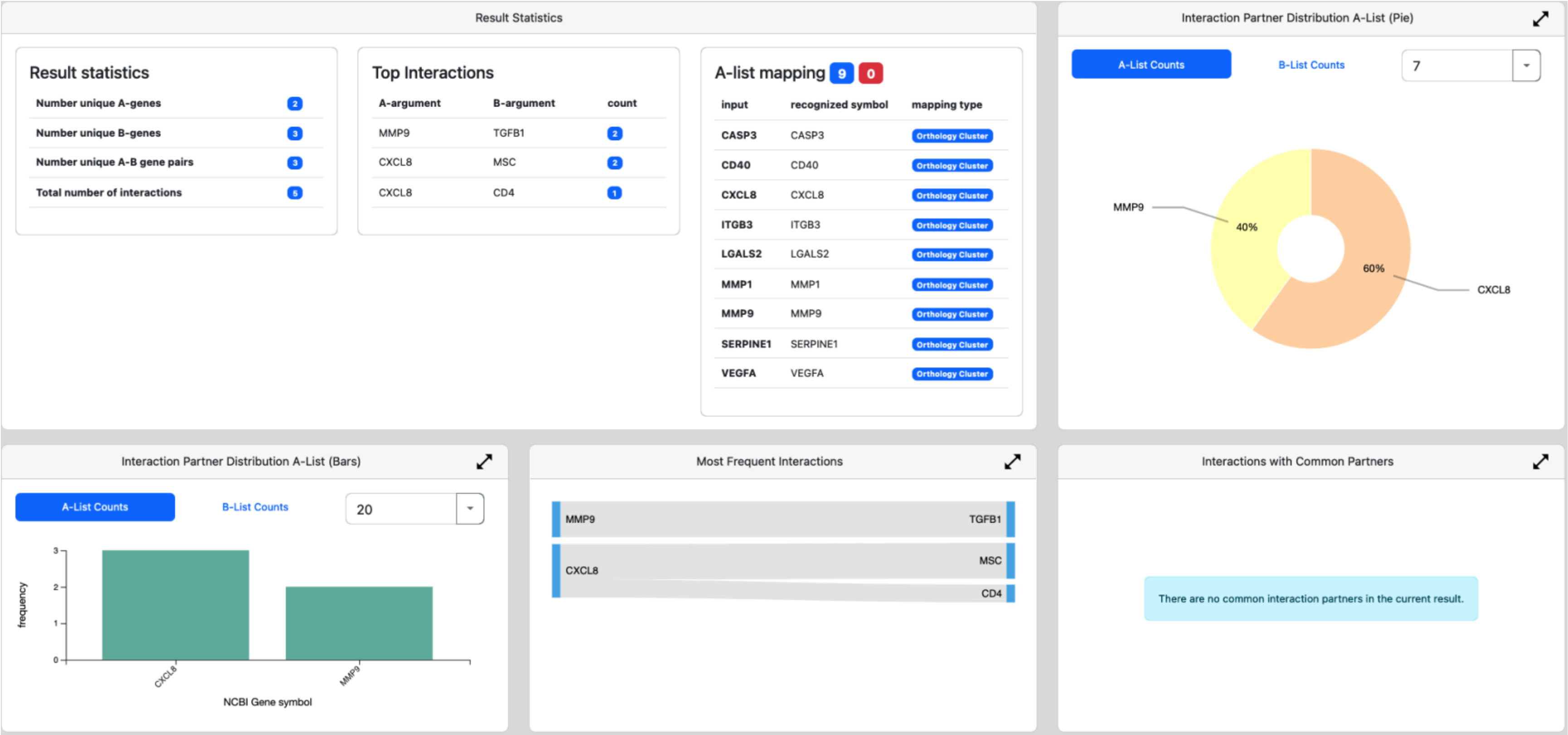
Result Statistics
Result statistics
Number unique A-genes 2
Number unique B-genes 3
Number unique A-B gene pairs 3
Total number of interactions 5
Top Interactions
A-argument B-argument count
MMP9 TGFB1 2
CXCL8 MSC 2
CXCL8 CD4 1
A-list mapping 9 0
input recognized symbol mapping type
CASP3 CASP3 Orthology Cluster
CD40 CD40 Orthology Cluster
CXCL8 CXCL8 Orthology Cluster
ITGB3 ITGB3 Orthology Cluster
LGALS2 LGALS2 Orthology Cluster
MMP1 MMP1 Orthology Cluster
MMP9 MMP9 Orthology Cluster
SERPINE1 SERPINE1 Orthology Cluster
VEGFA VEGFA Orthology Cluster
Interaction Partner Distribution A-List (Pie)
A-List Counts
B-List Counts
7
MMP9
40%
60%
CXCL8
Interaction Partner Distribution A-List (Bars)
A-List Counts
B-List Counts
20
frequency
3
2
1
0
CXCL8
MMP9
NCBI Gene symbol
Most Frequent Interactions
MMP9
TGFB1
CXCL8
MSC
CD4
Interactions with Common Partners
There are no common interaction partners in the current result.

Table View

Gene A Symbol	Gene B Symbol	Gene A Gene ID	Gene B Gene ID	Relation Types	Factuality	Fulltext Match Source	Document ID	Context
CXCL8	MSC	3576	9242	Positive_regulation	★★★	sentence	PMC6744626	The mechanistic link between inflammation and upregulation of SASP components was confirmed by experiments showing the upregulation of IL-6, IL-8, and MCP-1 in control MSC after exposure to TNF-alpha.<br>...cell transplantation.
CXCL8	CD4	3576	920	Binding	★★★	sentence	PMC7456414	Suggested predictors include neutrophils and CD4 T cells [], Serum Retinol-Binding Protein 4 [], C-reactive protein [] and 233 differential expressed genes in ALS monocytes, especially those related to inflammation (IL1B, IL8, FOSB, CXCL1, CXCL2) [].<br>Some studies indicated that human neural stem cells transplanted into a spinal cord...
CXCL8	MSC	3576	9242	Positive_regulation	★★★	sentence	PMC6744626	While the mechanistic link between inflammation and upregulation of SASP components was confirmed by experiments showing the upregulation of IL-6, IL-8, and MCP1 in control MSC after exposure to TNF-alpha, the observed differences between PE-MSC and NP-MSC could be secondary to hypertension and differences in gestational age.<br>Second, if autologous stem cell transplant is to be considered as a potential therapy...
Mmp9	Tgfb1	17395	21803	Regulation, Positive_regulation	★★★	sentence	PMC9287675	We also present evidence that mature neutrophils regulate macrophage inflammation via MMP9-mediated activation of TGF-β1.<br>Immune cells are reconstituted with different patterns post-HSCT, which provides...
Mmp9	Tgfb1	17395	21803	Positive_regulation	★★★	sentence	PMC9287675	Mechanistically, mature, but not immature, neutrophils regulated macrophage inflammation via MMP9-mediated activation of TGF-β1 (Figure 6).<br>...into severe sepsis, which remains a major cause of mortality for HSCT patients (

S6 Supplementary Material: Case Study 2 Demonstration

This document shows screenshots that complement the description of the case study on data from Weis et al. (2017) in the main manuscript.

The initial GePI query consisted of a paragraph-level context search:

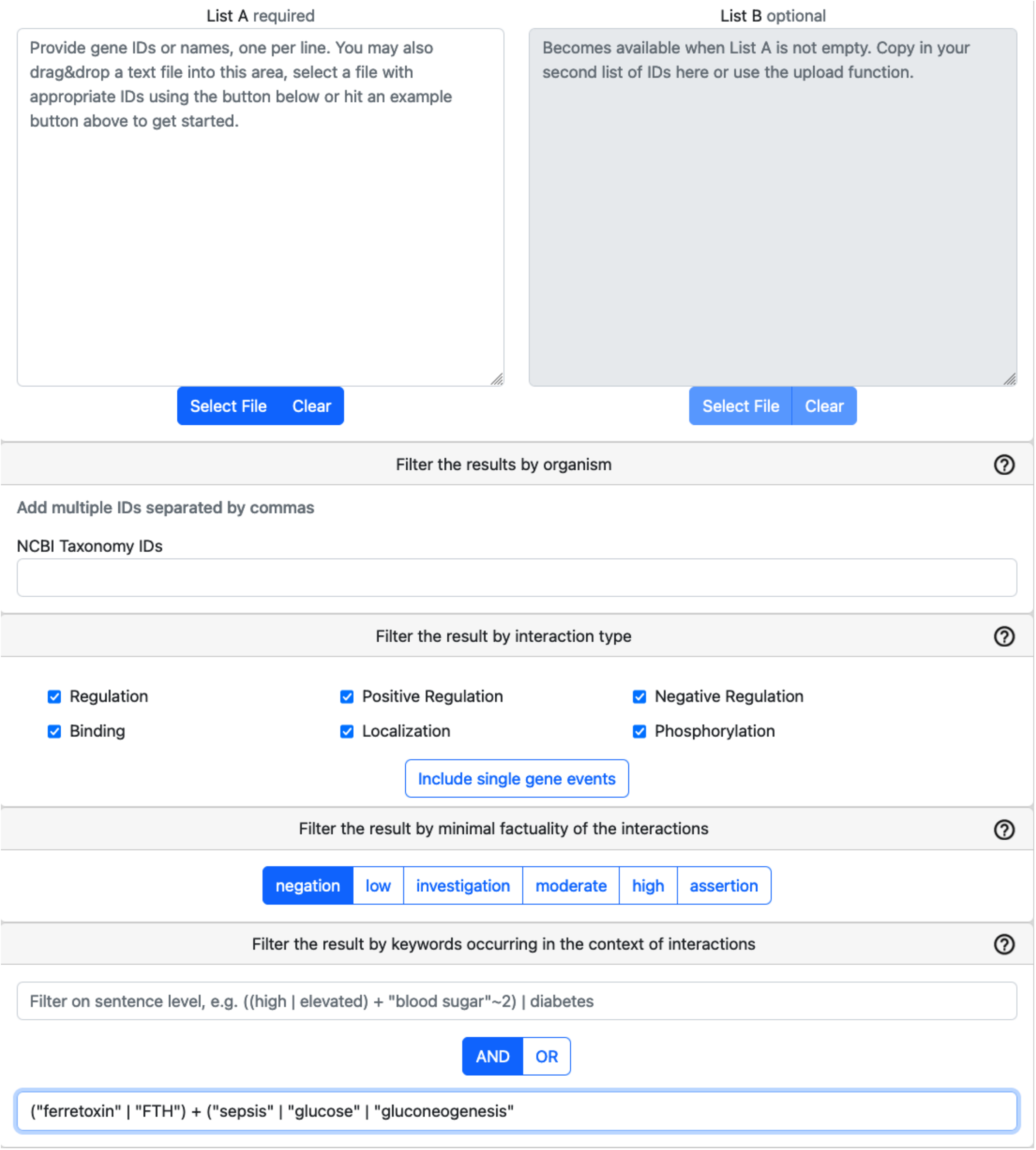

The result to this query is displayed in the following screenshots. The first shows statistics and visualizations regarding the found interaction partners and the interactions themselves.

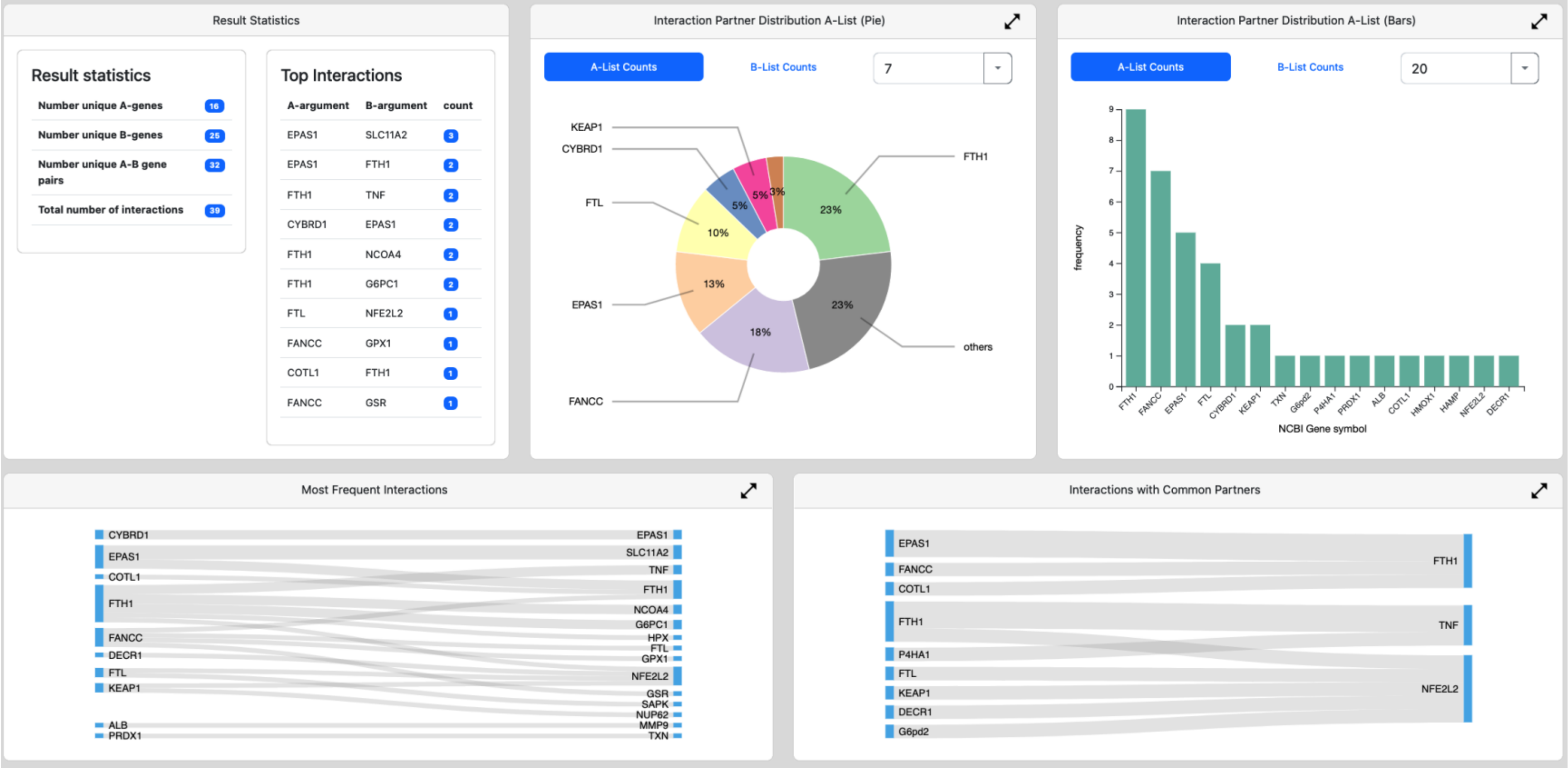

The result table lists all found interactions individually. The paragraph filter matches are given for each interaction in the context column, highlighted in deep blue.

Table View

1 2 3 4

Gene A Symbol	Gene B Symbol	Gene A Gene ID	Gene B Gene ID	Relation Types	Factuality	Fulltext Match Source	Document ID	Context
IRF3	Hmox1	3661	15368	Regulation, Positive_regulation	★★★	paragraph	PMC8347331	The relevance of myeloid HO-1 expression is highlighted in other studies where it was shown that myeloid HO-1 controls the activation of interferon-regulatory factor-3 after Toll-like receptor 3 or 4 stimulation, or viral infection, suggesting that HO-1 plays a critical function in innate immunity [].<br>...mice with global deletion of FtH [].
extracellular signal regulated kinase	FTL	FPLX:ERK	2512	Negative_regulation, Positive_regulation	★★★	paragraph	PMC6371952	We further identified that LPS mediated activation of MAPK pathways, specifically, JNK, and ERK were also reduced with FtL pre-treatment.<br>...the pathogenic sequelae of sepsis.
SAPK	FTL	FPLX:JNK	2512	Negative_regulation, Positive_regulation	★★★	paragraph	PMC6371952	We further identified that LPS mediated activation of MAPK pathways, specifically, JNK, and ERK were also reduced with FtL pre-treatment.<br>...the pathogenic sequelae of sepsis.
Hpx	Fth1	15458	14319	Negative_regulation, Regulation	☆☆☆	paragraph	PMC6958331	Hemopexin levels have been implicated as protective in a previous study with this model of sepsis [] but were not apparently affected by the loss of FtH in the myeloid cells (perhaps due in part to the wide range of individual values among the mice compared with those of Hp).<br>...and puncture model in mice that produces, as the gut contents leak, polymicrobial sepsis...
Rela	Ftl1	19697	14325	Gene_expression, Positive_regulation	★★★	paragraph	PMC6371952	Also, a recent study demonstrated that transfection of macrophages with FtL led to reduced nuclear accumulation of p65 subunit after LPS treatment ().<br>...in macrophages of FtHLysM−/− mice that underscores a compensatory mechanism to FtH...
Cotl1	Fth1	72042	14319	Regulation	★★★	paragraph	PMC6371952	Here, we asked whether myeloid cell specific FtH deletion modulates inflammation and organ injury in a commonly used and well-established model of poly-microbial sepsis, cecal ligation and puncture (CLP).<br>...and organ injury in a commonly used and well-established model of poly-microbial sepsis...
MMP9	ALB	4318	213	Gene_expression, Positive_regulation	★★★	paragraph	PMC8423709	Matrix metalloproteinase 9 (MMP9) served as positive control, as expression of MMP9 was previously demonstrated to be induced by albumin .<br>Expression of FTH-1 and FTL was higher upon FAC and GO /FAC stimulation, while FPN...
FTH1	GPX4	2495	2879	Regulation	★★★	paragraph	PMC8335735	Overall, these observations suggested that salusin-β contributed to HG-induced ferroptosis through regulation of genes for antioxidant system (GPX4 andSLC7A1) and iron metabolism regulation system (FTH-1 andTFR-1).<br>...ferroptosis through abnormal expressions ofGPX4, SLC7A11, FTH-1 andTFR-1.
Tnf	Fth1	21926	14319	Negative_regulation, Positive_regulation	★★★	paragraph	32430665	In vitro experiments showed that iron content was reduced, and lipopolysaccharide-induced Tnf-α (also known as Tnf) mRNA upregulation was inhibited in a macrophage cell line transfected with Fth siRNA.<br>Moreover, HFD administration impaired both glucose tolerance and insulin sensitivity...
Tnf	Fth1	21926	14319	Negative_regulation, Positive_regulation	★★★	paragraph	32430665	In vitro experiments showed that iron content was reduced, and lipopolysaccharide-induced Tnf-α (also known as Tnf) mRNA upregulation was inhibited in a macrophage cell line transfected with Fth siRNA.<br>Moreover, HFD administration impaired both glucose tolerance and insulin sensitivity...

The follow up search contained the cleaned result list of the initial result as a new closed search, i.e. both A- and B-list items are given. The paragraph-level filter “sepsis” was specified for contextualization:

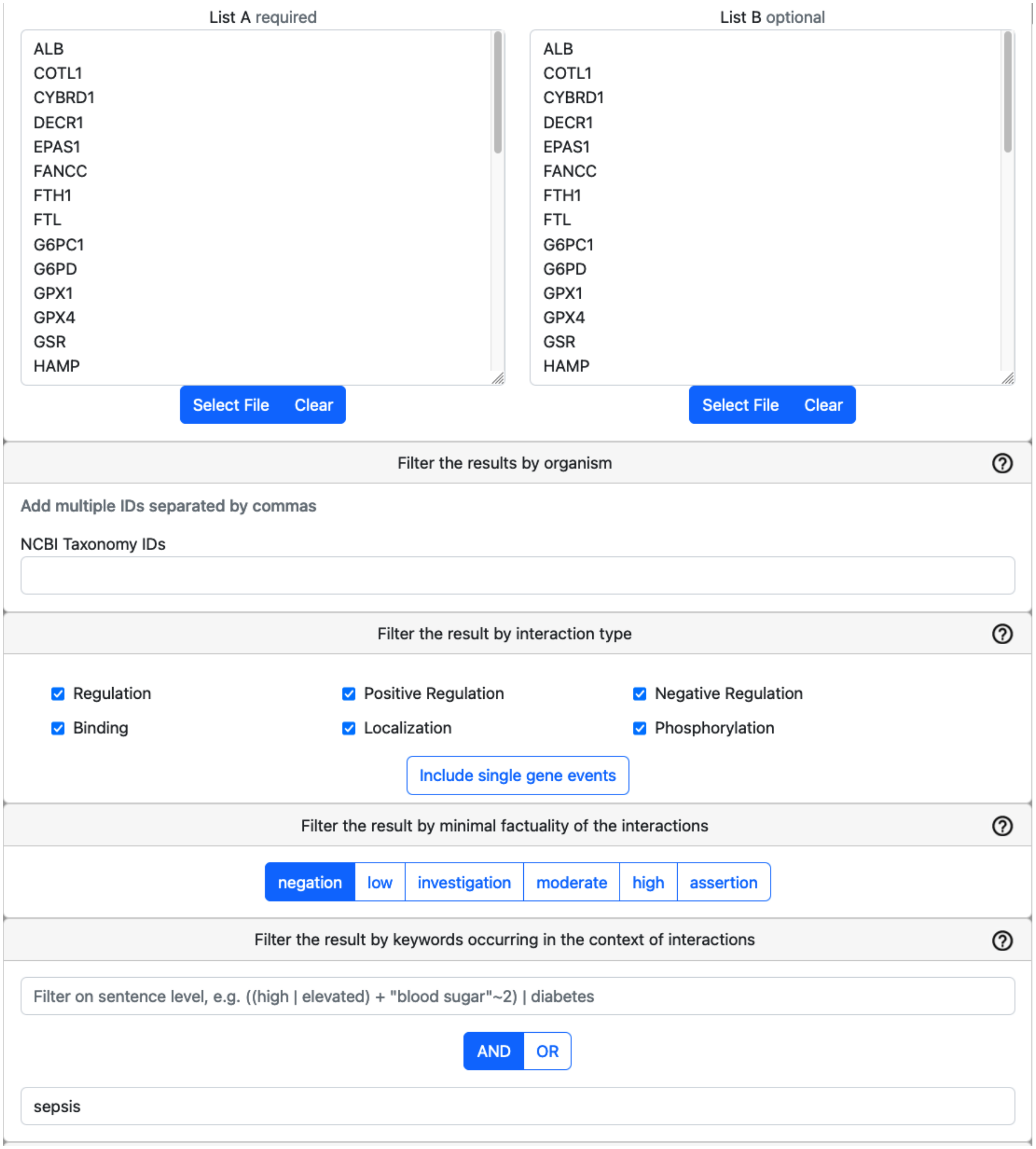

The result to the filtered query is shown below:

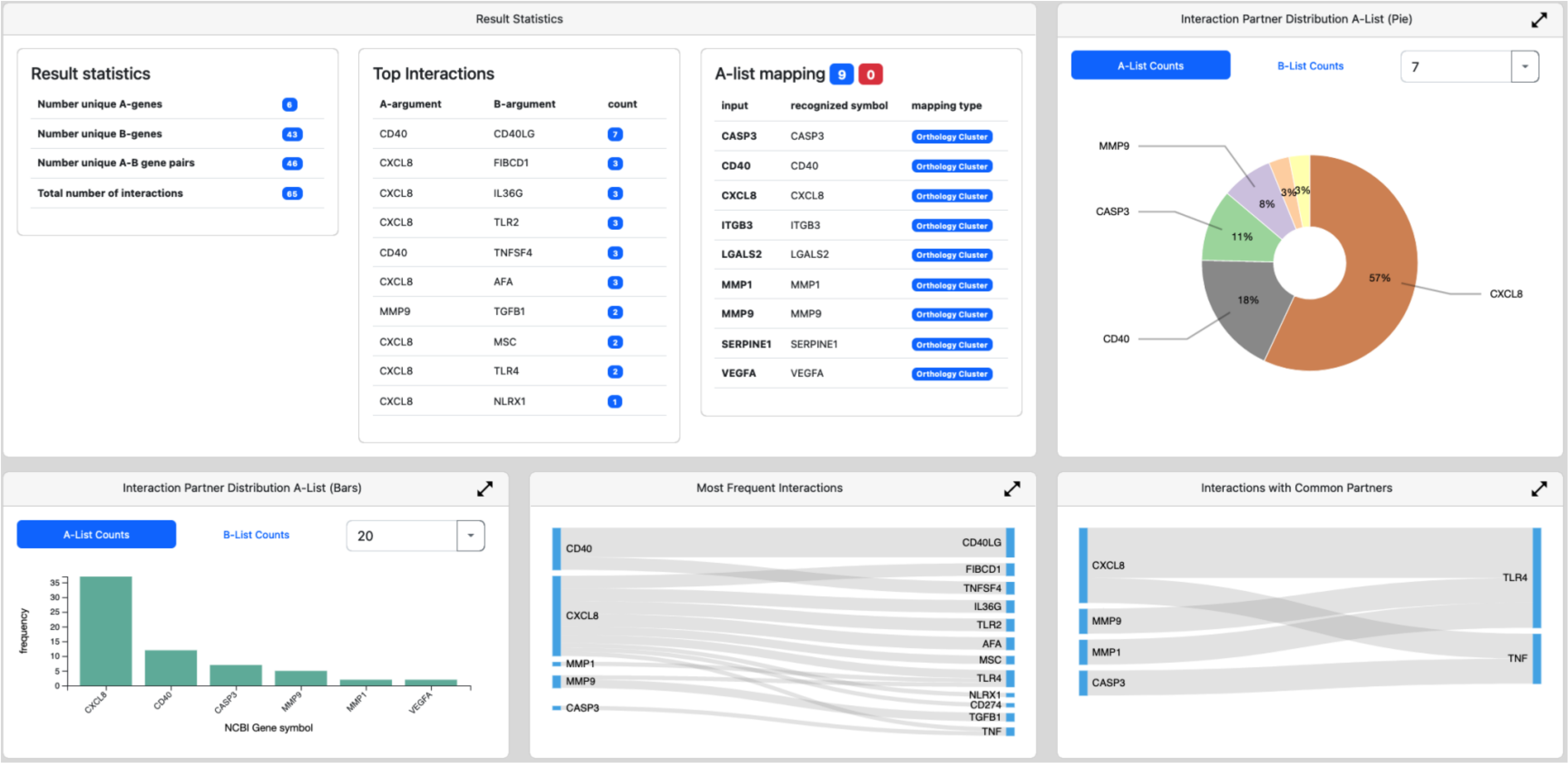
Result Statistics
Result statistics
Number unique A-genes 6
Number unique B-genes 43
Number unique A-B gene pairs 46
Total number of interactions 65
Top Interactions
A-argument B-argument count
CD40 CD40LG 7
CXCL8 FIBCD1 3
CXCL8 IL36G 3
CXCL8 TLR2 3
CD40 TNFSF4 3
CXCL8 AFA 3
MMP9 TGFB1 2
CXCL8 MSC 2
CXCL8 TLR4 2
CXCL8 NLRX1 1
A-list mapping 9 0
input recognized symbol mapping type
CASP3 CASP3 Orthology Cluster
CD40 CD40 Orthology Cluster
CXCL8 CXCL8 Orthology Cluster
ITGB3 ITGB3 Orthology Cluster
LGALS2 LGALS2 Orthology Cluster
MMP1 MMP1 Orthology Cluster
MMP9 MMP9 Orthology Cluster
SERPINE1 SERPINE1 Orthology Cluster
VEGFA VEGFA Orthology Cluster
Interaction Partner Distribution A-List (Pie)
A-List Counts
B-List Counts
7
MMP9
CASP3
CD40
CXCL8
57%
18%
11%
8%
3%
3%
Interaction Partner Distribution A-List (Bars)
A-List Counts
B-List Counts
20
frequency
NCBI Gene symbol
CXCL8
CD40
CASP3
MMP9
MMP1
VEGFA
Most Frequent Interactions
CD40
CXCL8
MMP1
MMP9
CASP3
CD40LG
FIBCD1
TNFSF4
IL36G
TLR2
AFA
MSC
TLR4
NLRX1
CD274
TGFB1
TNF
Interactions with Common Partners
CXCL8
MMP9
MMP1
CASP3
TLR4
TNF

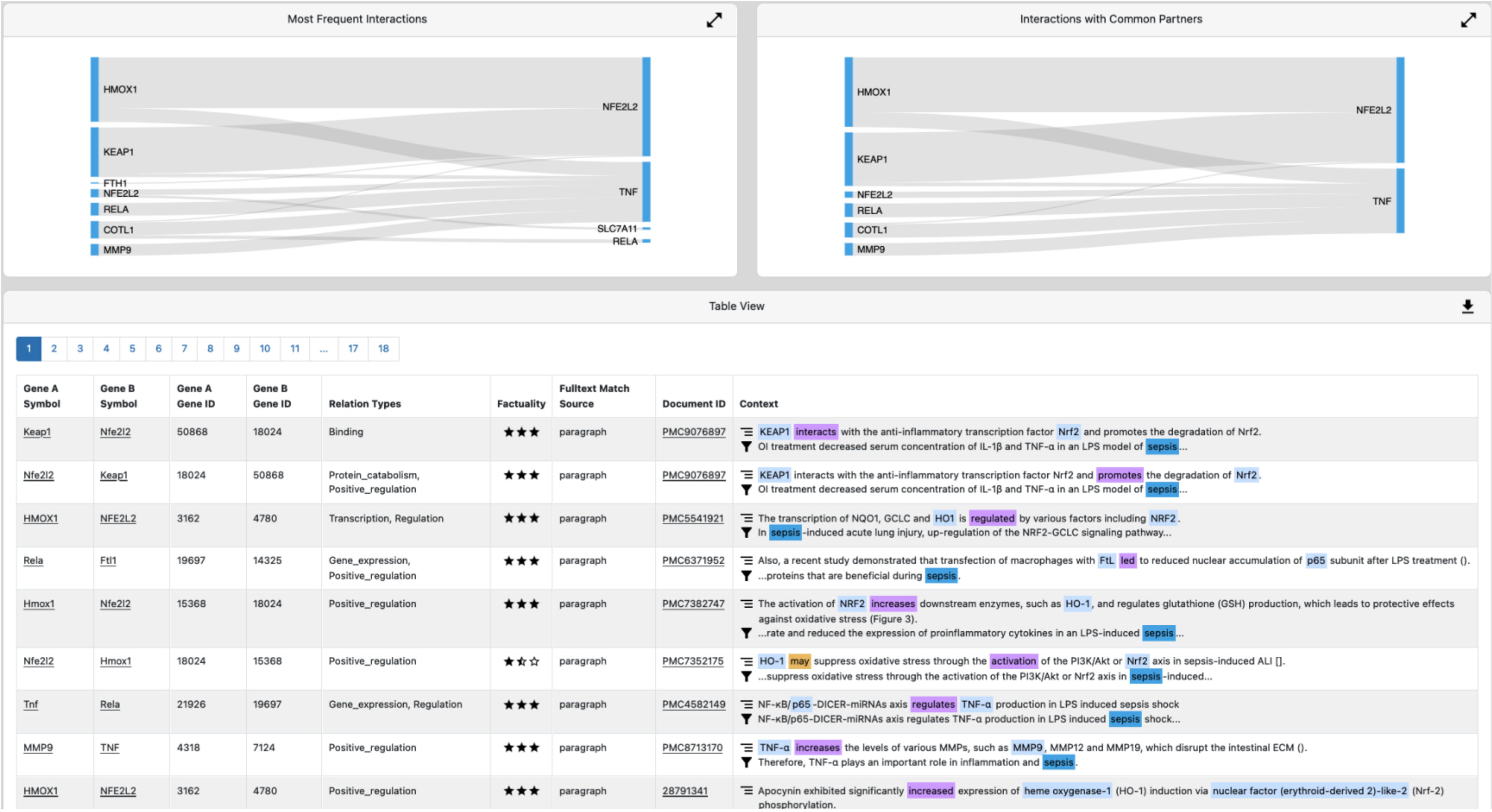

Gene A Symbol	Gene B Symbol	Gene A Gene ID	Gene B Gene ID	Relation Types	Factuality	Fulltext Match Source	Document ID	Context
Keap1	Nfe2l2	50868	18024	Binding	★★★	paragraph	PMC9076897	KEAP1 interacts with the anti-inflammatory transcription factor Nrf2 and promotes the degradation of Nrf2.<br>OI treatment decreased serum concentration of IL-1β and TNF-α in an LPS model of sepsis...
Nfe2l2	Keap1	18024	50868	Protein_catabolism, Positive_regulation	★★★	paragraph	PMC9076897	KEAP1 interacts with the anti-inflammatory transcription factor Nrf2 and promotes the degradation of Nrf2.<br>OI treatment decreased serum concentration of IL-1β and TNF-α in an LPS model of sepsis...
HMOX1	NFE2L2	3162	4780	Transcription, Regulation	★★★	paragraph	PMC5541921	The transcription of NQO1, GCLC and HO1 is regulated by various factors including NRF2.<br>In sepsis-induced acute lung injury, up-regulation of the NRF2-GCLC signaling pathway...
Rela	Ftl1	19697	14325	Gene_expression, Positive_regulation	★★★	paragraph	PMC6371952	Also, a recent study demonstrated that transfection of macrophages with FtL led to reduced nuclear accumulation of p65 subunit after LPS treatment ().<br>...proteins that are beneficial during sepsis.
Hmox1	Nfe2l2	15368	18024	Positive_regulation	★★★	paragraph	PMC7382747	The activation of NRF2 increases downstream enzymes, such as HO-1, and regulates glutathione (GSH) production, which leads to protective effects against oxidative stress (Figure 3).<br>...rate and reduced the expression of proinflammatory cytokines in an LPS-induced sepsis...
Nfe2l2	Hmox1	18024	15368	Positive_regulation	★★☆	paragraph	PMC7352175	HO-1 may suppress oxidative stress through the activation of the PI3K/Akt or Nrf2 axis in sepsis-induced ALI [].<br>...suppress oxidative stress through the activation of the PI3K/Akt or Nrf2 axis in sepsis-induced...
Tnf	Rela	21926	19697	Gene_expression, Regulation	★★★	paragraph	PMC4582149	NF-κB/p65-DICER-miRNAs axis regulates TNF-α production in LPS induced sepsis shock<br>NF-κB/p65-DICER-miRNAs axis regulates TNF-α production in LPS induced sepsis shock...
MMP9	TNF	4318	7124	Positive_regulation	★★★	paragraph	PMC8713170	TNF-α increases the levels of various MMPs, such as MMP9, MMP12 and MMP19, which disrupt the intestinal ECM ().<br>Therefore, TNF-α plays an important role in inflammation and sepsis.
HMOX1	NFE2L2	3162	4780	Positive_regulation	★★★	paragraph	28791341	Apocynin exhibited significantly increased expression of heme oxygenase-1 (HO-1) induction via nuclear factor (erythroid-derived 2)-like-2 (Nrf-2) phosphorylation.

To crosscheck the results for alternative verification, all identified genes taking part in at least one interaction were summarized and used as query against the STRING database (https://string-db.org) using *Mus musculus* as organism (29 April 2023).

The input query for STRING:

Alb
Cotl1
Decr1
Fth1
Ftl1
G6PD
Gpx4
GSR
Hamp
Hmox1
Hpx
IRF3
Keap1
Mapk9
Mmp9
Ncoa4
Nfe2l2
Nup62
Rela
Slc40a1
Slc7a11
Tfrc
Tnf
Txn

This produced the following network with STRING:

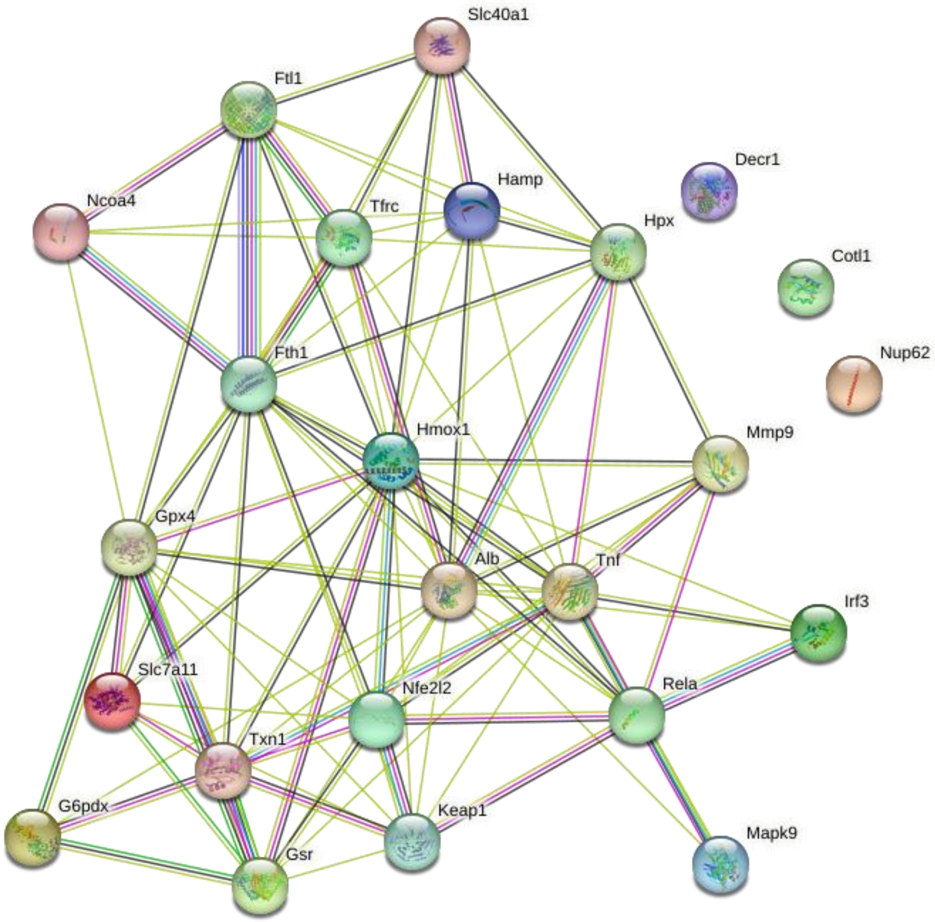

As can be seen from the picture above, with the exception of Cotl1, Decr1, and Nup62 all other entries showed multiple evidence for existing interactions between the mentioned genes with an average overall score of 0.66 (min: 0.4, max: 0.99).

GePI found 18 events for Cotl1 and 1 for Decr1 and Nup62 with the highest factuality score of assertion for each one. For further verification the respective publications and sentences are readily available for crosschecking from e.g. the website, or the downloadable excel spreadsheet.

References

Zoran T, Seelbinder B, White PL, Price JS, Kraus S, Kurzai O, Linde J, Häder A, Loeffler C, Grigoleit GU, Einsele H, Panagiotou G, Loeffler J, Schäuble S. Molecular Profiling Reveals Characteristic and Decisive Signatures in Patients after Allogeneic Stem Cell Transplantation Suffering from Invasive Pulmonary Aspergillosis. J Fungi (Basel). 2022 Feb 10;8(2):171. doi: 10.3390/jof8020171. PMID: 35205926; PMCID: PMC8880021.

Weis S, Carlos AR, Moita MR, Singh S, Blankenhaus B, Cardoso S, Larsen R, Rebelo S, Schäuble S, Del Barrio L, Mithieux G, Rajas F, Lindig S, Bauer M, Soares MP. Metabolic Adaptation Establishes Disease Tolerance to Sepsis. Cell. 2017 Jun 15;169(7):1263-1275.e14. doi: 10.1016/j.cell.2017.05.031. PMID: 28622511; PMCID: PMC5480394.
